# Predicting suspended sediment load in Peninsular Malaysia using support vector machine and deep learning algorithms

**DOI:** 10.1038/s41598-021-04419-w

**Published:** 2022-01-07

**Authors:** Yusuf Essam, Yuk Feng Huang, Ahmed H. Birima, Ali Najah Ahmed, Ahmed El-Shafie

**Affiliations:** 1 Department of Civil Engineering, College of Engineering, Universiti Tenaga Nasional, 43000 Selangor, Malaysia; 2 Department of Civil Engineering, Lee Kong Chian Faculty of Engineering and Science, Universiti Tunku Abdul Rahman, Selangor, Malaysia; 3 Department of Civil Engineering, College of Engineering, Qassim University, Unaizah, Saudi Arabia; 4 Department of Civil Engineering, College of Engineering, Institute of Energy Infrastructure (IEI), Universiti Tenaga Nasional, 43000 Selangor, Malaysia; 5 Department of Civil Engineering, Faculty of Engineering, University of Malaya, Kuala Lumpur, Malaysia

**Keywords:** Hydrology, Engineering

## Abstract

High loads of suspended sediments in rivers are known to cause detrimental effects to potable water sources, river water quality, irrigation activities, and dam or reservoir operations. For this reason, the study of suspended sediment load (SSL) prediction is important for monitoring and damage mitigation purposes. The present study tests and develops machine learning (ML) models, based on the support vector machine (SVM), artificial neural network (ANN) and long short-term memory (LSTM) algorithms, to predict SSL based on 11 different river data sets comprising of streamflow (SF) and SSL data obtained from the Malaysian Department of Irrigation and Drainage. The main objective of the present study is to propose a single model that is capable of accurately predicting SSLs for any river data set within Peninsular Malaysia. The ANN3 model, based on the ANN algorithm and input scenario 3 (inputs consisting of current-day SF, previous-day SF, and previous-day SSL), is determined as the best model in the present study as it produced the best predictive performance for 5 out of 11 of the tested data sets and obtained the highest average RM with a score of 2.64 when compared to the other tested models, indicating that it has the highest reliability to produce relatively high-accuracy SSL predictions for different data sets. Therefore, the ANN3 model is proposed as a universal model for the prediction of SSL within Peninsular Malaysia.

## Introduction

The background of the present study is first described in this section. This is followed by descriptions of the literature review, research gap, and contributions of the present study.

### Background

The conservation of river water quality is important for human civilization as river water often represents a source of potable water while also being used for irrigation purposes in many regions, including Peninsular Malaysia^1–4^. High suspended sediment loads (SSLs), which essentially comprise of tiny clay, silt, and sand particles, are known to have detrimental effects on the quality of river water as the sediments may act as transport mediums for pollutants and bacteria^5,6^. The pollutants include phosphorus and heavy metals namely zinc, mercury, and manganese. High suspended sediment loads (SSLs) also affect the ecosystems within rivers by reducing the survivability of aquatic plants as less sunlight is able to penetrate through the river water and be utilised for photosynthesis. History shows many instances of pollutions and disasters caused by unmonitored or unregulated SSL in Peninsular Malaysia and around the globe. In 2016, it was reported by Malaysia’s Natural Resources and Environment Minister that a major Malaysian river recorded a Nephelometric Turbidity Unit (NTU) of 6000, indicating a significantly high concentration of suspended sediments causing poor water quality. Recently in 2021, Sungai Pinang was reported to be polluted with sediments consisting of broken-down organic matter, causing the river to have a black appearance. This sediment-based pollution was a source of foul stench affecting a nearby food court and condominium within the vicinity of the Karpal Singh Drive. Also very recently in 2021, 305 lakes and rivers in Minnesota, United States were listed as too polluted to meet the required standards. Among the causes of high pollution were high sediment concentrations, which harmed fish as they struggled to find food due to high bacteria environments and algal blooms caused by eutrophication. Toxic algal blooms caused by sediments richly leached with nutrients such as phosphorus were also reported in 2018 at the St. Lucie River, Florida, United States, causing respiratory problems as well as irritation in the eyes and noses of the locals. Increased SSLs may also have an effect on dam and reservoir operations^1,7^. Dam inlets and channels can be obstructed by suspended sediments, while reservoir capacity may be reduced due to the settling of suspended sediments caused by relatively slow-moving water in the reservoir vicinity. Therefore, the ability to foresee the SSL within a particular river through predictions is especially important as a means to preserve the quality and supply of river water resources; to minimize or mitigate damages to the environment and hydrological structures namely dams and reservoirs; and to ensure the healthy continuity of hydrology-related activities such as irrigation^4,8^.

### Literature review

Traditionally, the sediment rating curve (SRC), which is a fitted relationship between suspended sediment concentration and river water discharge, has been utilised to assess trends and obtain predictions of SSLs, albeit having long response times and requiring a lot of information. However, a branch of artificial intelligence known as machine learning (ML), has been shown to effectively address these issues^5^ while producing more accurate SSL predictions compared to SRCs^1–3,9–11^. ML and deep learning, which is a more specialized version of ML typically consisting of neural networks, have also been used to solve important prediction problems within various fields. ML algorithms such as the decision tree (DT), random forest (RF), support vector machine (SVM) have been used for short-term water quality prediction to improve water management and pollution control, maize crop-yield prediction, and blockchain financial products earnings prediction to reduce concern of investors towards the risks and returns of financial products blockchain technology-based applications^12–14^; while deep learning algorithms such as the artificial neural network (ANN), long short-term memory (LSTM), and gated recurrent unit (GRU) have lately been utilized to solve more relatively complex problems such as the prediction of points-of-interest for purposes such as monitoring and maintaining public health following the coronavirus diseases (COVID-19), the prediction of greenhouse climate to ensure crop growth stability, and the prediction of health data with privacy reservation to combat the issue of missing data due to healthcare equipment failure and system updates^15–18^. In recent years, the artificial neural network (ANN) and support vector machine (SVM) algorithms have been shown to be among the most established and effective algorithms for application in the prediction of SSLs as shown by numerous existing literature^3,6,11,19–30^. Other than the ANN and SVM, other algorithms have also been studied for the purpose of SSL prediction. Meshram et al.^9^ studied the iterative classifier optimizer-based pace regression (ICO-PR) and iterative classifier optimizer-based random forest (ICO-RF) for SSL prediction in the Seonath River basin, India. It was shown that the ICO-RF is more accurate than the ICO-PR, and stand-alone PR and RF models. The study by Samadianfard et al.^31^ hybridized RF and multi-layer perceptron (MLP) with genetic algorithm (GA) and stochastic gradient descent (SGD) to produce four suspended sediment concentration (SSC) predictive algorithms namely GA-RF, GA-MLP, SGD-RF, and SGD-MLP. These algorithms were tested using data from the Minnesota and San Joaquin rivers; and it was determined that the GA-RF and GA-MLP models performed the best in predicting SSC for the Minnesota River, while the SGD-RF and SGD-MLP models were the most accurate for the San Joaquin River. Shadkani et al.^32^ used MLP, MLP-SGD, and gradient boosted tree (GBT), to predict SSL for the St. Louis and Chester stations along the Mississippi River, United States. It was found that the SGD optimization on the MLP resulted in more accurate SSL predictions, hence SGD-MLP was put forward as the most accurate model for SSL prediction. Hazarika et al.^5^ applied the coiflet wavelet-based large margin distribution machine-based regression (LDMR) and coiflet wavelet-based large margin distribution machine-based extreme learning machine (ELM) to predict SSL in the Tawang Chu River, India. The study showed that the two coiflet wavelet-based models produced better predictions compared to other tested models based on twin support vector regression (SVR), stand-alone LDMR, and stand-alone ELM. AlDahoul et al.^2^ studied the application of long short-term memory in predicting SSL at the Johor River basin, Malaysia. It was demonstrated that LSTM is capable of outperforming several other ML algorithms namely elastic net linear regression (ENLR), ANN, and extreme gradient boosting (XGB). The prediction of SSL using LSTM was also investigated in the study by Nourani and Behfar^33^, in which it was found that the LSTM-based models were superior to classical feed-forward neural networks in predicting SSL at the Mississippi River. The adaptive neuro-fuzzy inference system (ANFIS) was trialled with different membership functions to predict SSL for the Cumberland River, United States in the study by Babanezhad et al.^34^. ANFIS with the trimf membership function was found to produce the best predictive performance among the tested models, including ant colony optimization-based fuzzy inference system (ACOFIS). ANFIS was also hybridized with the bat algorithm (ANFIS-BA) in the study by Ehteram et al.^35^, in which it was found that ANFIS-BA was more reliable for SSL prediction in the Atrek River, Iran compared to other tested models namely ANFIS hybridized with whale algorithm (ANFIS-WA), and hybridized multi-feedforward neural network (MFNN) models with the BA and WA algorithms (MFNN-BA and MFNN-WA). The study by Azamathulla et al.^36^ applied genetic expression programming-based (GEP) models to predict SSLs in the Muda River, Langat River, and Kurau River in Malaysia. The GEP-based model was discovered to produce better predictive performances when compared to the other tested models which are ANFIS and a benchmark regression model. The dynamic evolving neural fuzzy inference system was studied by Adnan et al.^37^ for the prediction of SSL at two locations within China, namely Guangyuan and Beibei. DENFIS was shown to have a higher predictive accuracy compared to the other two models tested, which are ANFIS with fuzzy c-means clustering (ANFIS-FCM) and multivariate adaptive regression splines (MARS). However, in the study by Yilmaz et al.^1^, MARS was found to be capable of predicting SSL for the Çoruh River basin with the lowest error, compared to models based on the artificial bee colony (ABC) and teaching–learning based optimization. Tao et al.^8^ applied the radial basis M5Tree (RM5Ttree) to predict SSL for the Trenton hydrological station on the Delaware River, United States^8^. Results of the study showed that the RM5Tree model produced predictions with enhanced accuracy and outperformed the other tested models based on the response surface method (RSM), ANN and the classical M5Tree. Using the same data set applied in the study by Tao et al.^8^, Salih et al.^7^ used M5P, attribute classifier M5P (AS-M5P), M5Rule (M5R) and K Star (KS) models to predict SSL^7^. Different input scenarios of streamflow (SF) and SSL were used in this study, in which it was found that M5P was superior among the tested models. A hybrid version of the M5P, named bagging-M5P, was utilized by Khosravi et al.^38^ for SSL prediction in the Estero Morales River, Chile. The study showed bagging-M5P to be superior to the classical M5P, reduced error pruning tree (REPT), instance-based learning (IBK), and hybridized versions of the REPT model. Tabatabaei et al.^10^ predicted SSL using data from the Ramian hydrological station on the Ghorichay River, Iran by utilizing an SRC model optimized with the non-dominated sorting genetic algorithm II (NSGA-II), which increased prediction efficiency. In the study by Uca et al.^4^, multiple linear regression (MLR) and ANN were tested to predict SSL for the Jenderam catchment, Malaysia. The results demonstrated the capability of MLR in outperforming ANN with regards to SSL prediction accuracy.

### Research gap

A limitation that is present in majority of the aforementioned existing studies on SSL prediction is that most have focused on utilizing ML algorithms to develop predictive models for only one hydrological station or river, which means the models were developed based off of one data set. As the magnitude and behaviour of SSLs for each river is different, the suitability of certain ML algorithms for the task of SSL prediction may vary. Certain ML algorithms may be suitable and produce good SSL predictions for a hydrological station at a particular river but may not perform well in predicting SSLs for a different river, due to variance in anthropogenic and natural factors. In the case study of Peninsular Malaysia, existing studies have utilized ML algorithms, particularly ANN, MLR, LSTM, and GEP, to develop SSL predictive models^2,4,27–29,36^. Apart from the study by Azamathulla et al.^36^, all studies on SSL prediction within Peninsular Malaysia have focused on developing ML models solely based on data sets from single hydrological stations located in rivers such as Sungai Johor, Johor; Sungai Pari, Perak, Sungai Langat, Selangor, and the Jenderam catchment, Selangor. This creates a noteworthy research gap for the Peninsular Malaysia case study, as it is unknown whether there is a model or algorithm that is capable of producing accurate SSL predictions for multiple different rivers within the region. The present study contributes towards addressing this research gap through the development of predictive models for SSLs based on time series data sets of SF and SSLs from hydrological stations located along 11 different rivers throughout Peninsular Malaysia. The two established algorithms based on existing literature within the current field, namely SVM and ANN, were selected for utilization in the present study. In addition, LSTM was also chosen for the development of predictive models as it has recently been documented to have good ability in accurately predicting SSLs^2,33^, while also already performing well in other fields relating to flood forecasting, wind turbine fault diagnosis, rainfall-runoff modelling, building energy consumption forecasting, and drought forecasting^39–43^.

### Contributions

The present study was motivated by the aforementioned cases of SSL pollution in the Malaysian and American rivers, such as Sungai Pinang and St. Lucie River. Early anticipation and mitigation measures through the application of ML models could have played a significant role in reducing damages towards the local people and natural habitat. As there are many novel and advanced SSL-predicting models being developed in different study regions and demonstrated in scientific literature, practical adoption of ML predictive models for real-life application hydrological stations may not be straightforward due to the uncertainty of whether a selected ML model is able to replicate its good performance for different rivers with varying SSL behaviour and magnitude due to different anthropogenic and natural factors. Therefore, the scientific novelty of the present study is the selection and proposal of a single predictive model that is capable of producing SSL predictions of good accuracy for different rivers throughout Peninsular Malaysia. The major contribution of the present study is the testing and development of predictive ML models based on 3 different ML algorithms for hydrological stations on 11 different rivers throughout Peninsular Malaysia, in order to determine and propose a single ML model that is capable of predicting SSLs with high accuracy for multiple different rivers. Using time series data of SF and SSL for each river, SVM, ANN, and LSTM are tested to predict SSLs for each river using four different input scenarios. The performance of each model is evaluated using selected performance evaluation measures, namely mean absolute error (MAE), root mean squared error (RMSE) coefficient of determination (R^2^) and ranking mean (RM). The ML model that produces the best SSL predictions for the most rivers and obtains the best average RM is then proposed as a universal model that may be used for any specific case study within Peninsular Malaysia. The findings obtained in the present study may mainly be of interest to hydrological organizations looking for suitable or proven ML models for practical application within Peninsular Malaysia, as the models have been developed and tested using 11 different river data sets within the selected region. However, audiences from abroad may also take interest in the findings of the present study as the proposed SSL predictive model may possibly produce accurate SSL predictions for case studies in other regions around the world as well. The method of selecting the best SSL predictive ML model in the present study, which is by using performance evaluation measures to determine the model that produces the best SSL predictions for the most rivers and obtains the best average RM, may also be a point of interest for a wider audience regardless of geographic location. The rest of the present study is organized as follows: Sect. 2 describes the materials and methods used to carry out the present study. Section 3 reports and discusses the results of the present study. Section 4 concludes the overall study.

## Materials and methods

In this section, the materials and methods employed in the process of predicting SSL for the 11 selected rivers within Peninsular Malaysia are explained. Important information regarding the location and data of case study, model development process, ML algorithms, data pre-processing, and performance evaluation measures are described.

### Location and data of case study

Peninsular Malaysia represents the western region of Malaysia comprising of 13 states and 2 federal territories. It encompasses a total area of 132,265 km^2^, which is about 40% of the total area of Malaysia; and is located just North of the equator. Peninsular Malaysia has approximately 1235 river basins^44^, with Sungai Pahang representing the longest river in the region at 459 km in length. In the present study, raw data in the form of daily average SF and daily total SSL were obtained from the Water Resources Management and Hydrology Division of the Malaysian Department of Irrigation and Drainage for different rivers within 11 states in Peninsular Malaysia. Based on the volume and continuity of the available data; and the relevance of the rivers to their respective state, one river is selected per state for the purpose of the present study. Information on the selected rivers for each state; identification and location of the hydrological measuring stations; and the duration of data provided by the respective station for each selected river is shown in Table 1.

**Table 1:** Information on selected rivers’ data for each state.

State	River	Streamflow station no	Suspended sediment station no	Latitude	Longitude	Data duration
Johor	Sungai Johor	1,737,451	1,737,551	01°46′50"N	103°44′45"E	1978 to 1998
Kedah	Sungai Muda	5,605,410	5,606,510	05°36′35"N	100°37′35"E	1976 to 2009
Kelantan	Sungai Kelantan	5,721,442	5,721,542	05°45′45"N	102°09′00"E	1980 to 1997
Melaka	Sungai Melaka	2,322,413	2,322,513	02°20′35"N	102°15′10"E	1979 to 2004
Negeri Sembilan	Sungai Kepis	2,723,401	2,723,501	02°42′20"N	102°21′20"E	1980 to 1995
Pahang	Sungai Pahang	3,527,410	3,527,510	03°30′45"N	102°45′30"E	1988 to 2009
Perak	Sungai Perak	4,809,443	4,809,553	04°49′10"N	100°57′55"E	1977 to 1995
Perlis	Sungai Arau	6,503,401	6,503,501	06°30′10"N	100°21′05"E	1986 to 1995
Selangor	Sungai Selangor	3,414,421	3,414,521	03°24′10"N	101°26′35"E	1976 to 2001
Terengganu	Sungai Dungun	4,832,441	4,832,541	04°50′35"N	103°12′15"E	1977 to 1996
F.T. of Kuala Lumpur	Sungai Klang	3,116,430	3,116,530	03°08′20"N	101°41′50"E	2010

### Model development process

**Figure 1:**
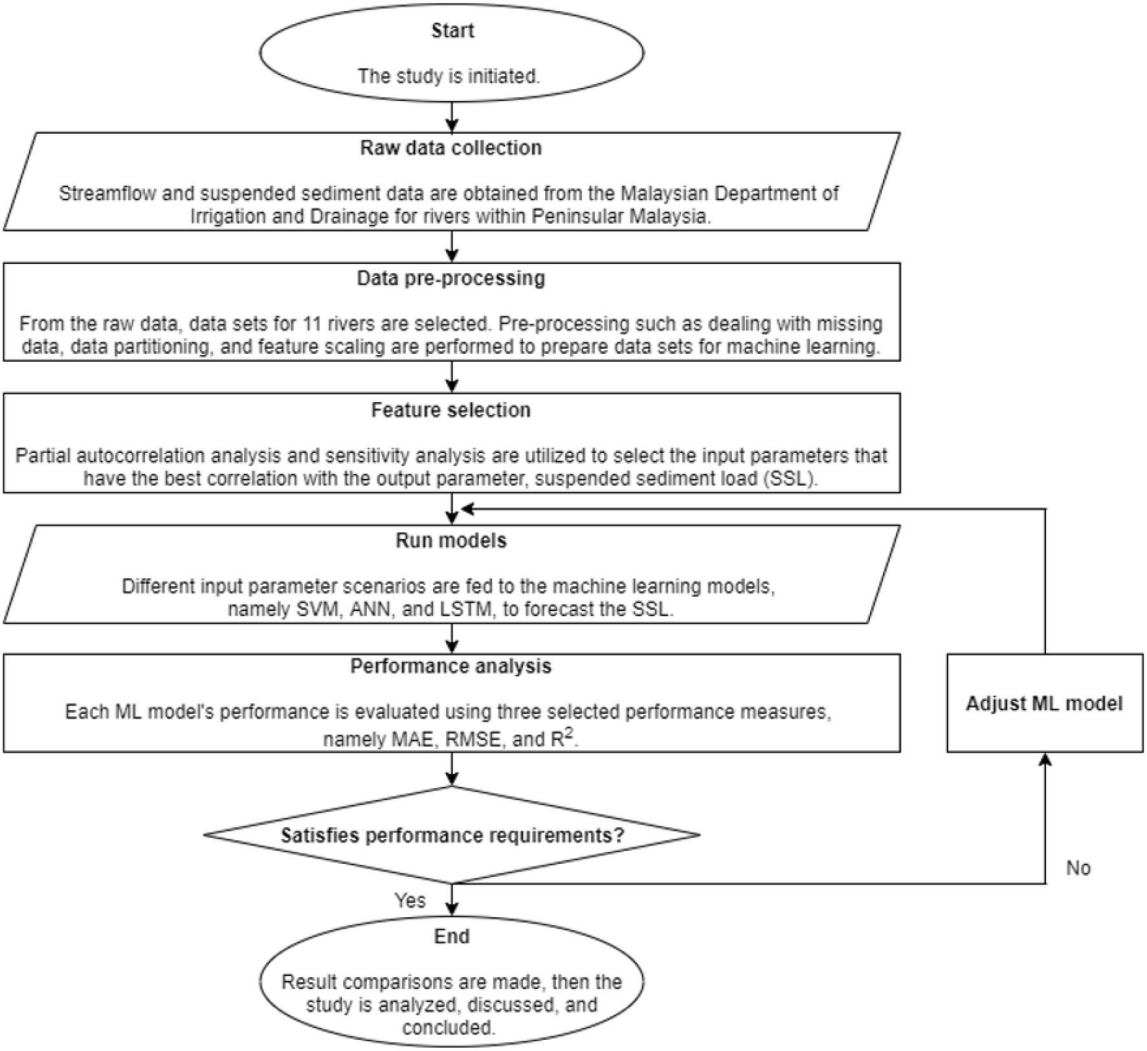
The model development process employed in the present study.

The model development process in the present study consists of raw data collection, data pre-processing, feature selection, model prediction, and performance analysis. This is illustrated as shown in Fig. 1.

### Machine learning algorithms

Three ML algorithms were chosen for SSL prediction in the present study, which are SVM, and two deep learning algorithms namely ANN and LSTM. The SVM and ANN algorithms are considered established in the current field as they have been shown to produce good SSL predictions by many existing literature^3,6,11,19–30^, while the LSTM algorithm has recently been found to produce good SSL-predicting performances in recent studies^2,33^ and has already shown good performances in several other studies within different fields^39–43^. The Python programming language was selected for the development of SSL predictive models as it easy to comprehend and command, while also having good library support. The system specifications used to develop the predictive models in the present study are detailed in Table 2.

**Table 2:** System specifications used to train and test ML models.

Parameter	Specification
Programming language	Python 3.7.12
ML libraries	Scikit-learn 1.0 (for SVM) TensorFlow 2.6.0 (for ANN, LSTM)
Notebook environment	Jupyter (hosted by Colaboratory)
Central processing unit (CPU)	Intel® Core™ i7-6700HQ CPU @ 2.60 GHz
Random access memory (RAM)	16.0 GB
System type	64-bit operating system, × 64 based processor

#### Support vector machine (SVM)

The SVM, also known as the decision support system, was proposed by Vapnik^45^. It represents a kernel-based algorithm that is abundantly used for pattern classification, function approximation, and also regression analyses^24,26^, especially in hydrological and time series simulations^21^. The version of SVM used to solve regression analyses is typically known as support vector regression (SVR), which is utilized to predict SSL in the present study. SVR generally works by estimating the learning data through the definition of a function, and determining the linear separation function to enable realistic results that reflect its statistical learning theory^24^. SVM is advantageous in the way that it has less tendency to overfit, has good ability to generalize, can simultaneously minimize estimation errors, and linearly separate inputs in a mapped high dimensional feature space^26^. However, SVM is sensitive to noise, hence its predictive ability reduces when the utilized data set is significantly noisy^46–48^. Its predictive performance also reduces with larger data sets due to an increase in training time^48^. SVM will also underperform in instances where there are fewer training data samples in comparison to the number of features for each data point. According to Buyukyildiz & Kumcu^24^, the SVM function is given by:

1 $$f\left(x\right)=\sum_{i=1}^{N}{(\alpha }_{i}-{\alpha }_{i}^{*})K(x,z)+{b}_{i}$$

where ${(\alpha }_{i}-{\alpha }_{i}^{*})$ is the Lagrange multiplier, $K(x,z)$ is the kernel function inside the multiplier, and ${b}_{i}$ is bias.

The main SVR hyperparameter that is tuned before running the SVR models is the kernel function. According to Himanshu et al.^26^ and Rahgoshay et al.^21^, among the four kernel functions that can be utilized namely radial basis functions (RBF), linear, polynomial, and sigmoid, RBF represents the best function to be used because of its good ability in handling complicated parameter space^26^. Through trial and error, it was indeed found that RBF gave the best results for SSL prediction in the present study. All other unmentioned SVR hyperparameters were left as their default values given that good predictions were obtained. The SVR hyperparameter tuning is shown in Table 3; while the time and space complexity of SVR are as follows:

2 $$Training\, time\, complexity=O({n}^{2})$$

3 $$Training \,space\, complexity=O(k)$$

where *n* is the number of data points and *k* is the number of support vectors.

**Table 3:** Hyperparameter tuning for SVR algorithm.

Hyperparameter tuning of SVR algorithm	
Kernel function	RBF

#### Artificial neural network (ANN)

The ANN is a non-linear data processing mathematical algorithm that is able to connect multiple input variable to produce one or more output variables^8,28^. It is based on the biological functioning of the neural connections within the human brain^19^. This algorithm typically comprises of three layers, which are the input layer, hidden layer, and output layer. The hidden layer may consist of either one or multiple layers, and functions to make sense of a multidimensional expansion of the input layer^19^. The architecture of an ANN is made up of units called neurons, also known as nodes^8,19,28^. One of the drawbacks of ANN is that this algorithm can be computationally expensive and highly dependent on hardware capability^49–51^. The ANN requires processing power parallel with its structure, hence adequate processors are needed for the models to be trained with realistic and efficient training durations. In addition, there does not exist a specific set of rules to determine the ANN structure during model development or coding. Therefore, a suitable ANN architecture is to be achieved with model development experience and processes of trial-and-error. Other than that, the ANN has a black-box nature that limits its ability to pin-point causal relationships between variables and a particular output; and may overfit during training due to model interaction or non-linearity^50,51^. According to several studies^8,19,28^, the mathematical model of an ANN may be represented by:

4 $${y}_{i}=f\left(\sum_{i=1}^{N}{\omega }_{ij}{x}_{i}+{b}_{j}\right)$$

where ${y}_{i}$ is the output variable, *N* is the number of neurons, ${\omega }_{ij}$ is the weight connecting the *j*^*th*^ neuron and the *i*^*th*^ neuron, ${x}_{i}$ is the input vector, *b*_*j*_ is the bias of the *jth* neuron, and *f* is the activation function.

As mentioned by Mustafa et al.^28^, there is no certain rule for selecting the number of neurons in the hidden layer. Therefore, this hyperparameter must be selected through trial-and-error. Through testing, it was found that 2 hidden layers with 6 neurons in each layer was good for SSL prediction in the present study as it provided good adaptability in producing good results for the 11 different river data sets. Other than the number of neurons and number of hidden layers, different number of epochs, batch numbers, training algorithms, and activation functions were tested to find the best possible ANN architecture capable of adapting to the 11 different river data sets. The best ANN architecture found is shown in Table 4. Other unmentioned hyperparameters including initialiser, regulariser, and constraints, are remained default as good predictions are obtained. The time and space complexity of the ANN are as follows:

5 $$Training\, time\, complexity=O(ne(ij+jk+kl))$$

6 $$Training \,space \,complexity=O(z)$$

where n is the number of data points, e is the number of epochs, i is the number of input layer neurons, j is the number of second layer neurons, k is the number of third layer neurons, l is the number of output layer neurons, and z is the total number of neurons.

**Table 4:** Hyperparameter tuning for ANN algorithm.

Hyperparameter tuning of ANN algorithm	
Number of hidden layers	2
Number of neurons in each hidden layer	6
Number of epochs	100
Early call-back function	When validation loss does not improve after 50 epochs
Batch number	32
Training algorithm	Adam
Activation function	ReLU
Loss function	MSE

**Figure 2:**
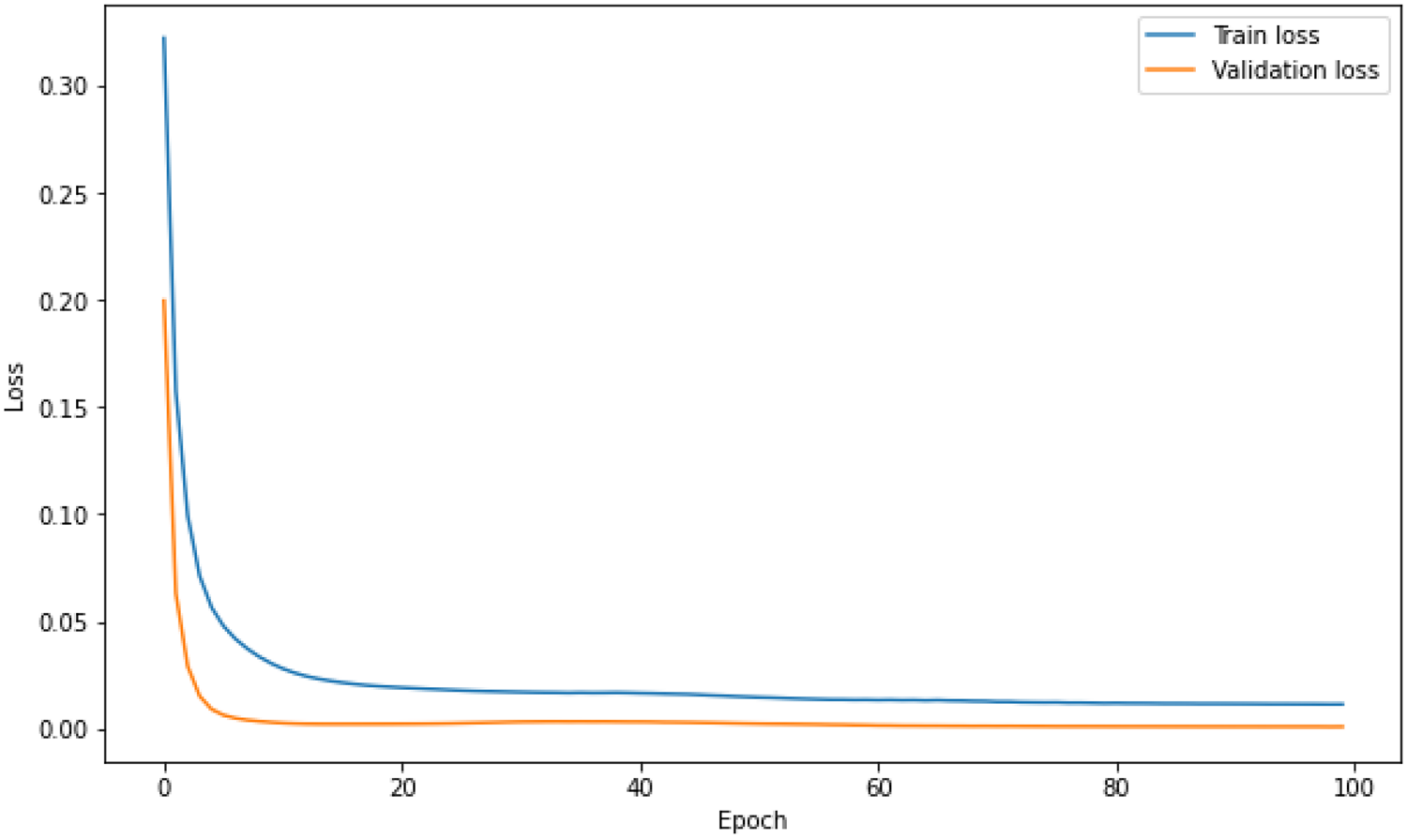
Train and validation loss vs epochs for ANN3 model training process.

The train and validation loss vs epochs graphs are produced during each of the ANN models’ training process. This is to ensure through graphical observation that the losses reduce and converge, while also to verify that overfitting does not occur during training. As a sample, the losses vs epochs graph for the best performing ANN model (ANN3) for the Johor data set is shown in Fig. 2.

#### Long short-term memory (LSTM)

The LSTM is an advanced version of the recurrent neural networks (RNN) that addresses issues with conventional RNNs relating to gradient vanishing and explosion. This algorithm boasts memory cells and control gates, which together are able to collect and store information^2,39^. The four control gates are the input gate, update gate, forget gate, and output gate^42^. They enable the writing, updating, forgetting, and reading of the information forwarded from the memory cells^42^. Through the operation of the control gates, the LSTM is hence able to minimise errors by retaining relevant information and forgetting irrelevant information as needed. Similar to the ANN and deep learning ML algorithms in general, the LSTM requires high computational power to train and develop predictive models^52,53^. The high memory-bandwidth needed given the presence of linear layers in each cell may reduce the hardware efficiency of this algorithm. Depending on the LSTM architecture and difficulty of the problem at hand, the LSTM may also take significantly long to train and develop^54^. Additionally, the LSTM is prone to overfitting^55,56^, hence needing dropout regularization and early call-back mechanisms to reduce overfitting effects. According to Guo et al.^57^, the LSTM output is generally computed through function:

7 $${h}_{t}={o}_{t}{\odot \mathrm{tanh}(C}_{t})$$

where *h*_*t*_ is the output, *o*_*t*_ is the output gate, $\odot$ is the Hadamard product, and *C*_t_ is the cell status value at time *t*.

Similar to ANNs, LSTMs also have hidden layers that are occupied by neurons. A trial-and-error process must be carried out to determine an optimal number of hidden layers and neurons in each hidden layer. Through testing, it was determined that 2 hidden layers with 50 neurons in each hidden layer was able to provide the best possible overall SSL predictions for the 11 different river data sets tested in the present study. The number of epochs, step number, batch number, training algorithm, dropout regularization on each hidden layer, activation function, and recurrent activation function were also experimented with in order to determine the best LSTM architecture for the present study, which is detailed in Table 5. Other unmentioned hyperparameters including initialiser, regulariser, and constraints, are remained default as good predictions are obtained. Given that LSTMs are local in time and space^58^, the overall computational complexity of an LSTM for each time step can be described by:

8 $$Overall \,computational \,complexity=O(w)$$

where *w* is the number of weights.

**Table 5:** Hyperparameter tuning of LSTM algorithm.

Hyperparameter tuning of LSTM algorithm	
Number of hidden layers	2
Number of neurons in each hidden layer	50
Number of epochs	100
Early call-back function	When validation loss does not improve after 20 epochs
Step number	7
Batch number	32
Training algorithm	Adam
Dropout regularization on each hidden layer	0.2
Activation function	tanh
Recurrent activation function	sigmoid
Loss function	MSE

**Figure 3:**
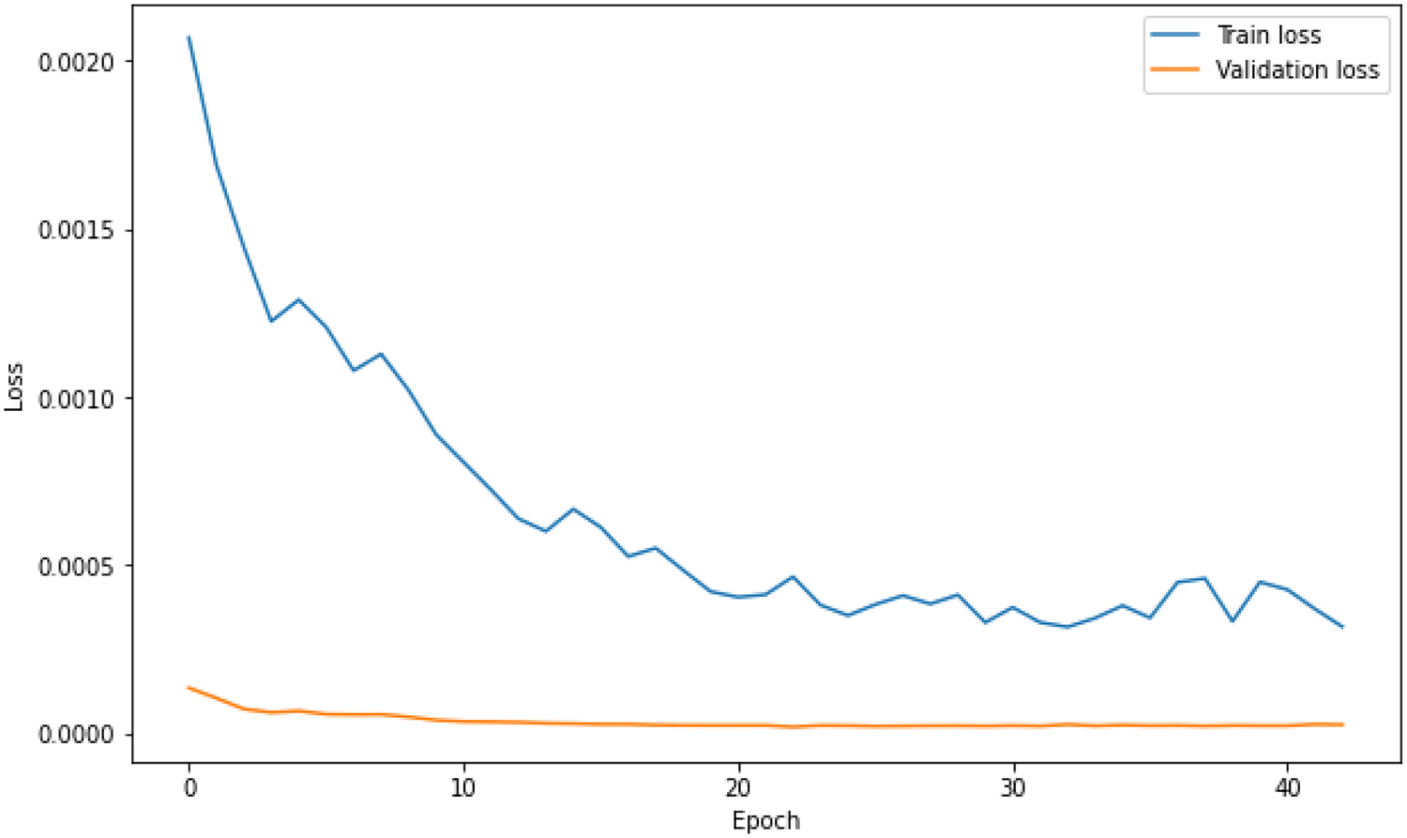
Train and validation loss vs epochs for LSTM3 model training process.

The train and validation loss vs epochs graphs are produced during each of the LSTM models’ training process. This is to ensure through graphical observation that the losses reduce and converge, while also to verify that overfitting does not occur during training. As a sample, the losses vs epochs graph for the best performing LSTM model (LSTM3) for the Johor data set is shown in Fig. 3.

### Data pre-processing

The pre-processing steps performed on the raw data sets for the selected 11 rivers obtained from the Malaysian Department of Irrigation and Drainage are detailed in this section. The data pre-processing steps include file merging and preparation, imputation of missing data, data partitioning, and feature scaling. These steps are performed to prepare the data sets to be fed to the ML algorithms for training and testing of models.

#### File merging and preparation

The SSL and SF data sets for each selected river were obtained separately in .txt file format from the Malaysian Department of Irrigation and Drainage. Given that there are 11 selected rivers, a total of 22 .txt files (11 SSL .txt files and 11 SF .txt files) were collected. For each river, a .csv file was then prepared by merging data from the SSL and SF .txt files of each corresponding river. The SF and SSL data in the .csv files were arranged into three columns, headed by ‘Date’, ‘SF(t)’, and ‘SSL(t)’. SF(t) denotes the SF at time t or the current SF, while SSL(t) denotes the SSL at time t or the current SSL. Unimportant information that readily came with the raw data sets were removed.

#### Missing data

**Figure 4:**
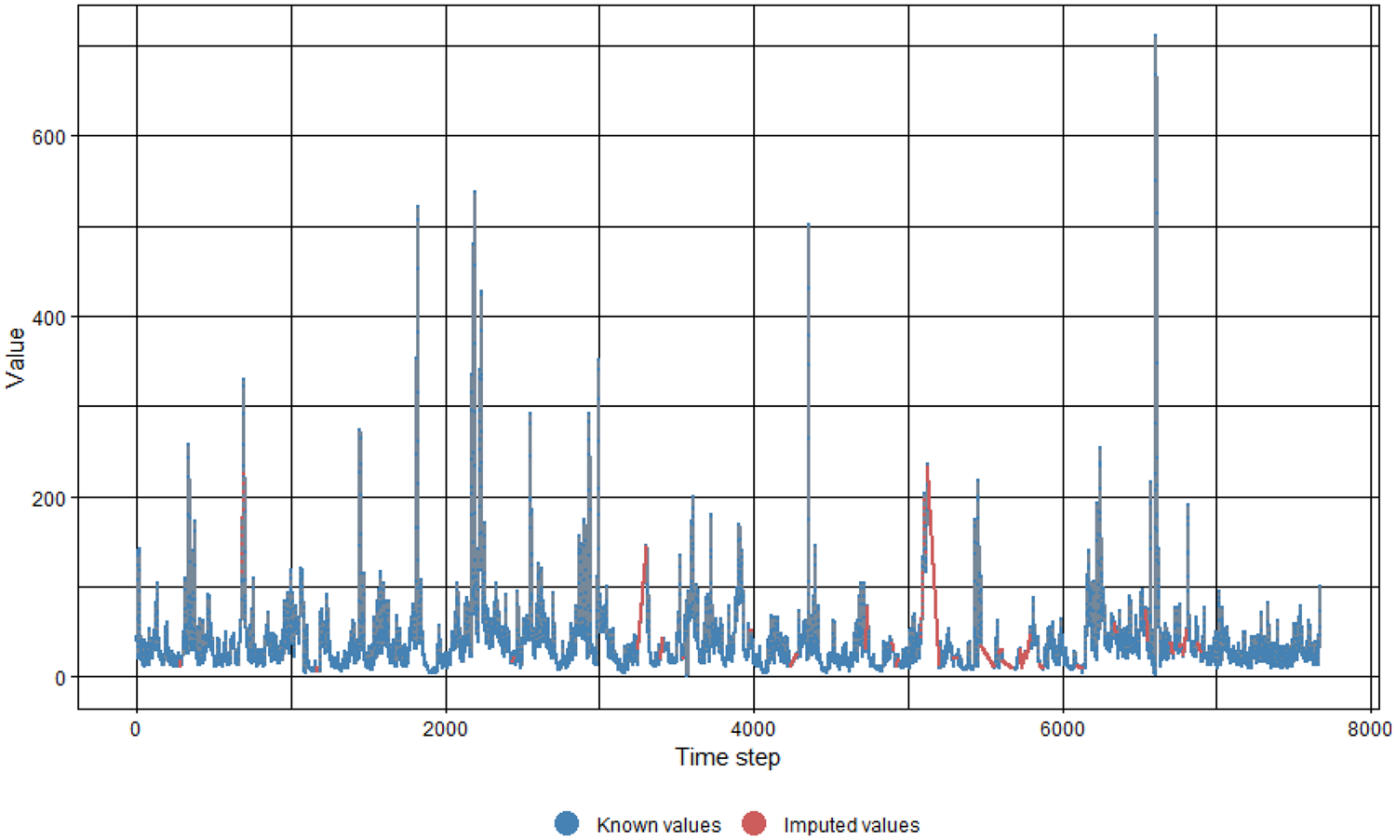
SF imputed values for Johor data set. (SF values in units of m^3^/s, time step in units of day).

**Figure 5:**
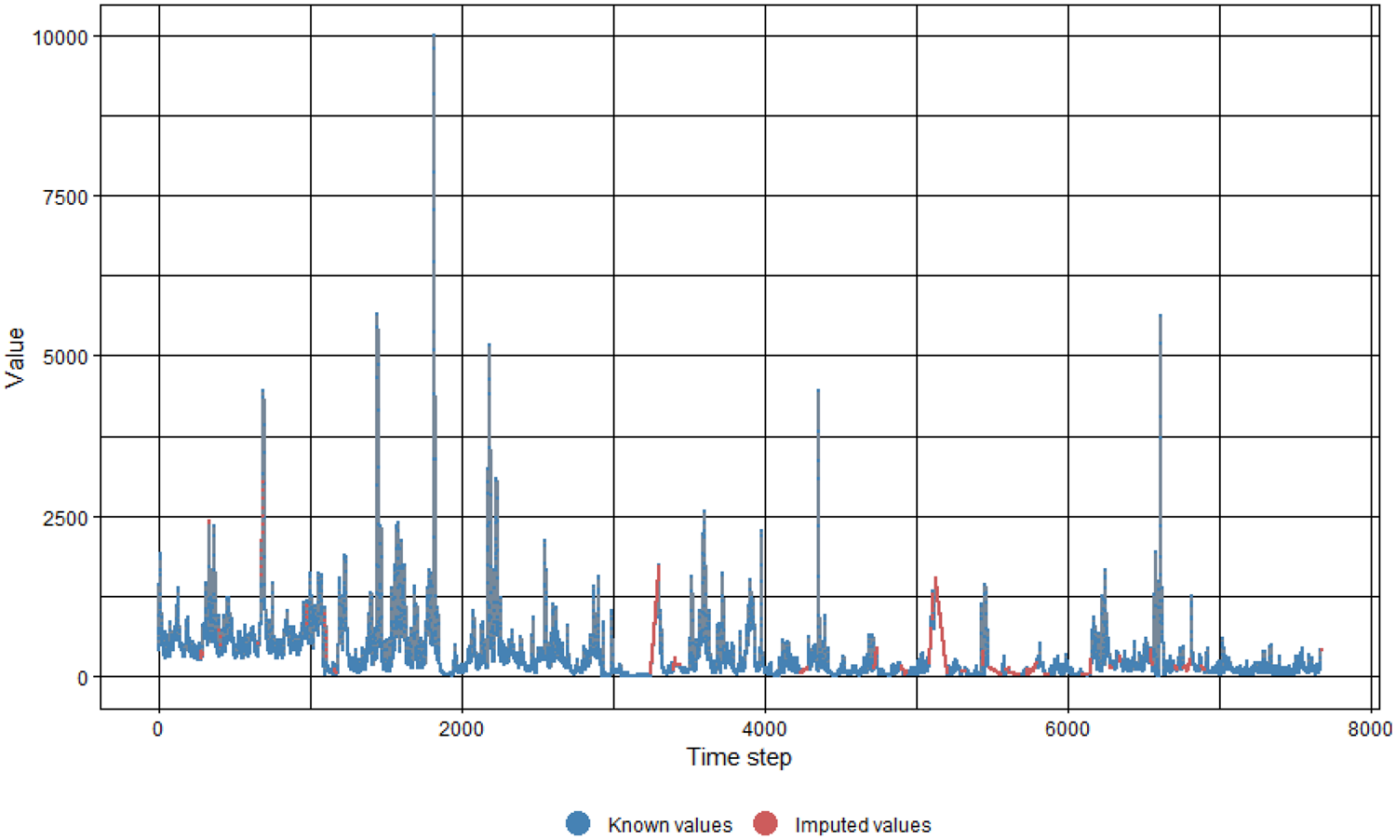
SSL imputed values for Johor data set. (SSL values in units of ton/day, time step in units of day).

The raw SF and SSL time-series data sets contained missing values which needed to be dealt with before proceeding with model training. This is because most ML algorithms produce errors when they encounter missing values within a data set. In the study field of suspended sediments, it is demonstrated by previous studies that imputation using interpolation has been utilized to fill in for missing or unavailable data values with likely and reasonable values^59–62^. The imputeTS package, developed by Moritz & Bartz-Beielstein^63^ and available in the RStudio environment, was utilized for imputation in the present study. Linear interpolation was adopted to inhabit the sections within the data sets in which values are missing. As a sample, the outcome of the imputation process for missing values in the Johor data set for SF and SSL is shown in Figs. 4 and 5 respectively.

#### Data partitioning

Data partitioning is applied to the data sets to segregate the daily SF and SSL data for each river into a training set and a test set. The training set is utilized to develop and equip the ML models with the ability to make SSL predictions, while the test set is used to evaluate the ability and accuracy of the ML models’ predictions with the help of selected performance measures. With reference to the study by Kannangara et al.^64^, an optimum ratio for training to testing is found to be 80:20. Existing studies on SSL predictions have also shown to use and produce good results using a training data to testing data ratio of 80:20^2,19,36^. Therefore, in the present study, 80% of each river’s data set is taken for training while the remaining 20% is used for testing. Using this ratio, each river’s data set is partitioned accordingly with similar statistical properties as shown in Table 6. Additionally, it is worth to note that for the deep learning algorithms (ANN, LSTM), 20% of the training set is used for validation.

**Table 6:** Data partitioning for each river’s data set.

River data set	Total duration	Training set	Test set
Sungai Johor, Johor	1^st^ January 1978 to 31^st^ December 1998	1^st^ January 1978 to 26^th^ October 1994	27^th^ October 1994 to 31^st^ December 1998
Sungai Muda, Kedah	1^st^ January 1976 to 31^st^ December 2009	1^st^ January 1976 to 22^nd^ March 2003	23^rd^ March 2003 to 31^st^ December 2009
Sungai Kelantan, Kelantan	1^st^ January 1980 to 31^st^ December 1997	1^st^ January 1980 to 2^nd^ June 1994	3^rd^ June 1994 to 31^st^ December 1997
Sungai Melaka, Melaka	1^st^ January 1979 to 31^st^ December 2004	1^st^ January 1979 to 27^th^ October 1999	28^th^ October 1999 to 31^st^ December 2004
Sungai Kepis, Negeri Sembilan	1^st^ January 1980 to 31^st^ December 1995	1^st^ January 1980 to 25^th^ October 1992	26^th^ October 1992 to 31^st^ December 1995
Sungai Pahang, Pahang	1^st^ January 1988 to 31^st^ December 2009	1^st^ January 1988 to 14^th^ August 2005	15^th^ August 2005 to 31^st^ December 2009
Sungai Perak, Perak	1^st^ January 1977 to 31^st^ December 1995	1^st^ January 1977 to 20^th^ March 1992	21^st^ March 1992 to 31^st^ December 1995
Sungai Arau, Perlis	1^st^ January 1986 to 31^st^ December 1995	1^st^ January 1986 to 7^th^ January 1994	8^th^ January 1994 to 31^st^ December 1995
Sungai Selangor, Selangor	1^st^ January 1976 to 31^st^ December 2001	1^st^ January 1976 to 26^th^ October 1996	27^th^ October 1996 to 31^st^ December 2001
Sungai Dungun, Terengganu	1^st^ January 1977 to 31^st^ December 1996	1^st^ January 1977 to 7^th^ January 1993	8^th^ January 1993 to 31^st^ December 1996
Sungai Klang, F.T. of Kuala Lumpur	1^st^ January 2010 to 31^st^ December 2010	1^st^ January 2010 to 26^th^ October 2010	27^th^ October 2010 to 31^st^ December 2010

#### Feature scaling

Feature scaling is required to be performed on all data sets as both SVM and the deep learning algorithms used in the present study are sensitive to data scales. This data pre-processing step may be carried out through either normalisation or standardisation depending on the type of ML algorithm to be used. Feature scaling ensures that all data variables are accurately weighted to ensure fast convergence and error minimisation during training^4,28^. In the present study, standardisation is applied on the data sets before training the SVM models, while normalisation is used before training the ANN and LSTM models. Both the input and output data are scaled before training and testing the models, and it is ensured that the ML models’ outputs are inverse transformed back into their original scales before evaluation using the selected performance measures.

### Feature selection

Feature selection is essentially the process of selecting input parameters to be used for model training. For the present study, in addition to daily SSL data for the 11 selected rivers, SF data has also been provided. Existing studies have described SF to significantly affect SSL, as larger river discharges enables the transport of sediment through the water body at a higher rate, hence increasing the SSL magnitude^65–68^. The present study has hence utilised both daily average SF and daily total SSL data to make SSL predictions, as previous studies have produced good SSL predictions by using these inputs^5,11,24,37^. A statistical analysis on the daily SF and SSL data for each of the 11 selected rivers is shown in Table 7.

**Table 7:** Statistical analysis of daily SF and SSL data for the 11 selected rivers. (SF is in units of m^3^/s, SSL is in units of ton/day).

River data set	Data	Mean	Median	Mode	Standard dev	Min	Max	Count
Sungai Johor, Johor	SF	40.0	28.7	15.9	43.4	0.5	709.7	7670
	SSL	338.0	170.0	70.0	507.4	1.0	9985.0	7670
Sungai Muda, Kedah	SF	87.1	58.0	26.0	88.3	3.0	1160.0	12,419
	SSL	428.7	79.0	5.0	1061.9	0.0	9736.0	12,419
Sungai Kelantan, Kelantan	SF	495.7	364.2	509.3	587.1	81.7	9775.1	6575
	SSL	368.3	101.0	4.0	744.6	1.0	7820.0	6575
Sungai Melaka, Melaka	SF	5.8	3.3	1.4	7.8	0.0	119.9	9497
	SSL	167.2	25.0	1.0	500.2	0.0	9473.0	9497
Sungai Kepis, Negeri Sembilan	SF	0.5	0.2	0.1	1.7	0.0	65.3	5844
	SSL	4.3	0.0	0.0	85.2	0.0	6247.0	5844
Sungai Pahang, Pahang	SF	683.5	520.6	497.3	540.5	133.0	6285.3	8036
	SSL	8.8	5.0	4.0	24.9	0.0	705.0	8036
Sungai Perak, Perak	SF	219.6	212.0	250.0	109.7	19.0	988.0	6939
	SSL	805.0	409.0	42.0	1076.2	0.0	9971.0	6939
Sungai Arau, Perlis	SF	0.7	0.0	0.0	1.6	0.0	23.0	3652
	SSL	6.1	1.0	1.0	17.5	0.0	318.0	3652
Sungai Selangor, Selangor	SF	53.9	44.0	34.7	36.7	2.3	313.9	9497
	SSL	790.0	338.0	9.0	1062.1	2.0	9178.0	9497
Sungai Dungun, Terengganu	SF	124.5	78.2	50.0	185.1	9.4	3178.8	7305
	SSL	325.3	91.9	13.0	604.2	0.0	8526.0	7305
Sungai Klang, F.T. of Kuala Lumpur	SF	0.5	19.7	20.8	9.6	10.3	105.6	365
	SSL	814.6	755.0	789.0	597.0	224.0	6006.0	365

**Figure 6:**
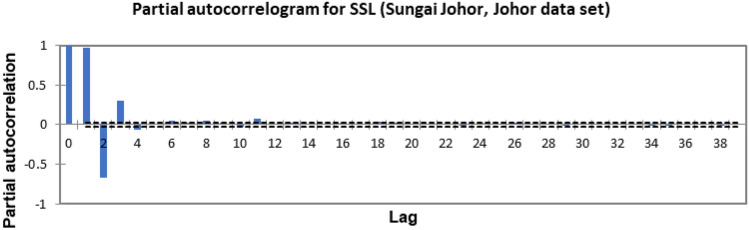
Partial autocorrelogram for SSL (Sungai Johor, Johor data set).

**Figure 7:**
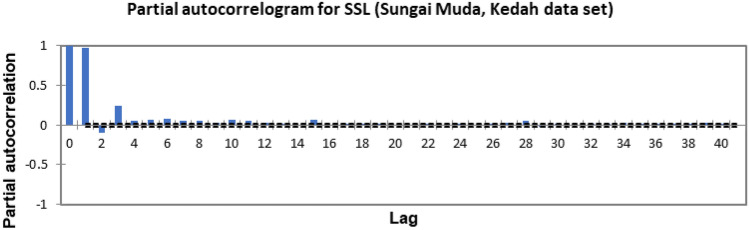
Partial autocorrelogram for SSL (Sungai Muda, Kedah data set).

**Figure 8:**
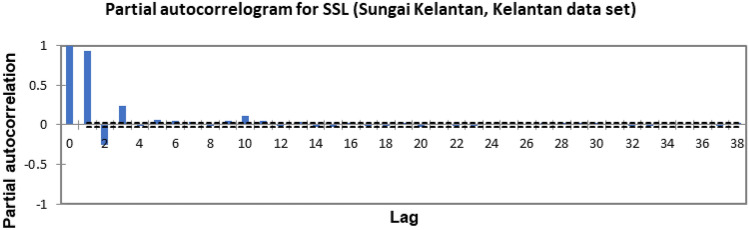
Partial autocorrelogram for SSL (Sungai Kelantan, Kelantan data set).

**Figure 9:**
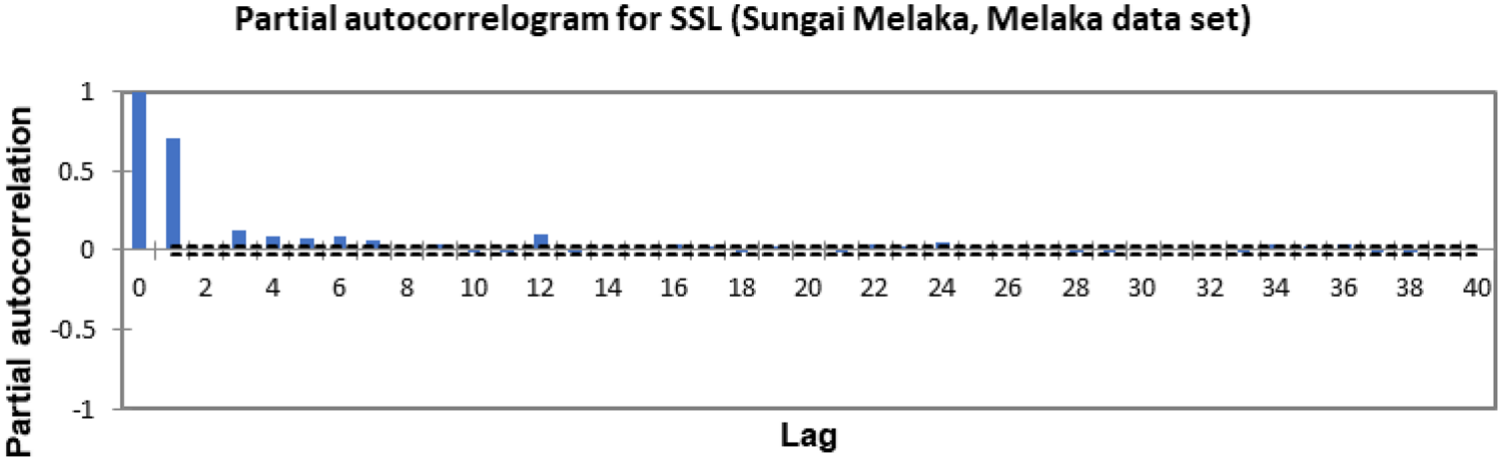
Partial autocorrelogram for SSL (Sungai Melaka, Melaka data set).

**Figure 10:**
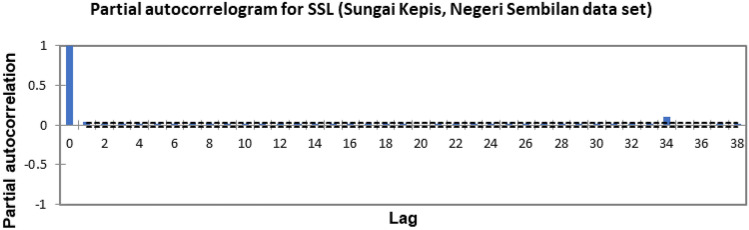
Partial autocorrelogram for SSL (Sungai Kepis, Negeri Sembilan data set).

**Figure 11:**
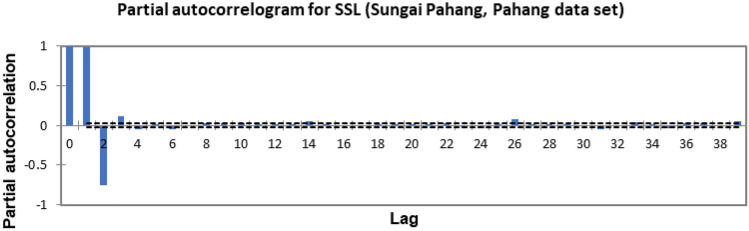
Partial autocorrelogram for SSL (Sungai Pahang, Pahang data set).

**Figure 12:**
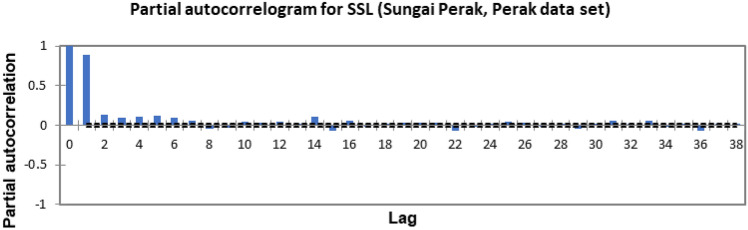
Partial autocorrelogram for SSL (Sungai Perak, Perak data set).

**Figure 13:**
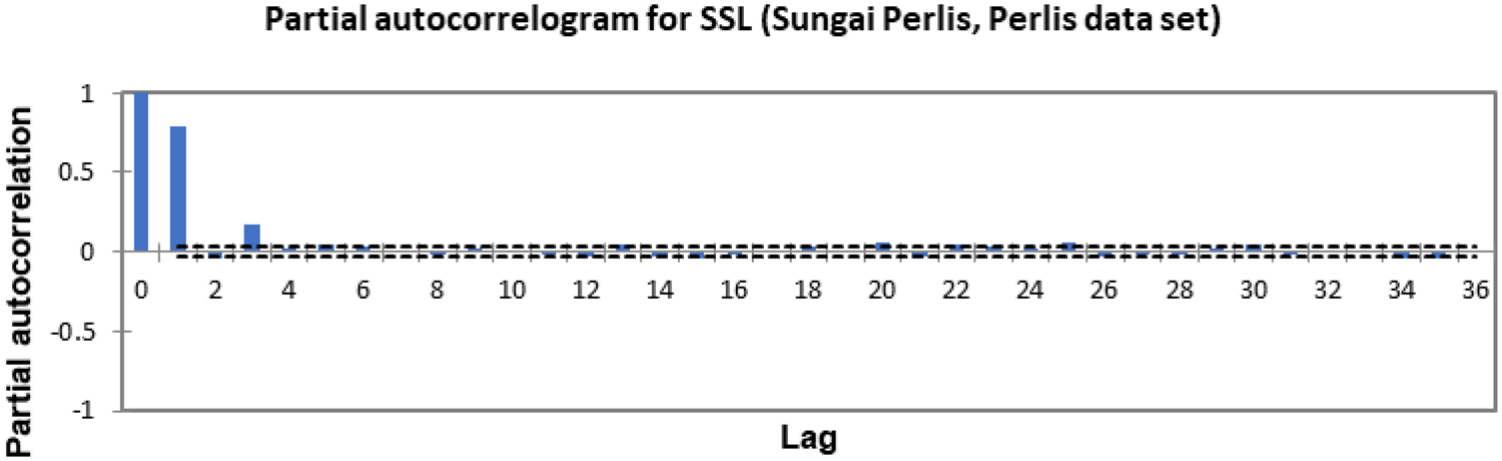
Partial autocorrelogram for SSL (Sungai Perlis, Perlis data set).

**Figure 14:**
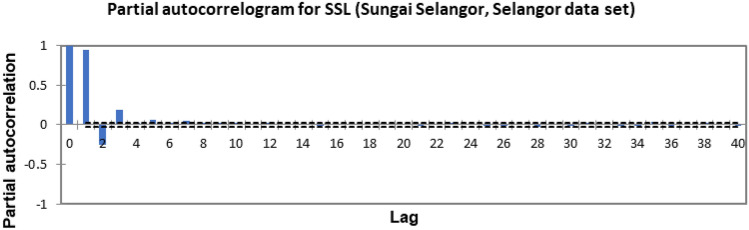
Partial autocorrelogram for SSL (Sungai Selangor, Selangor data set).

**Figure 15:**
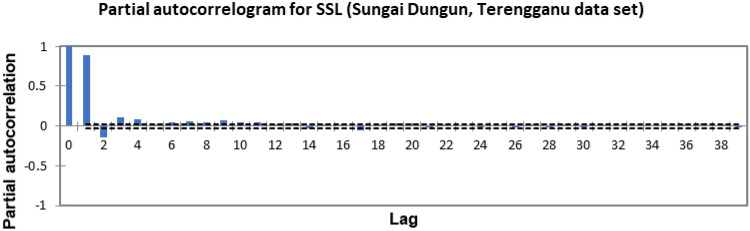
Partial autocorrelogram for SSL (Sungai Dungun, Terengganu data set).

**Figure 16:**
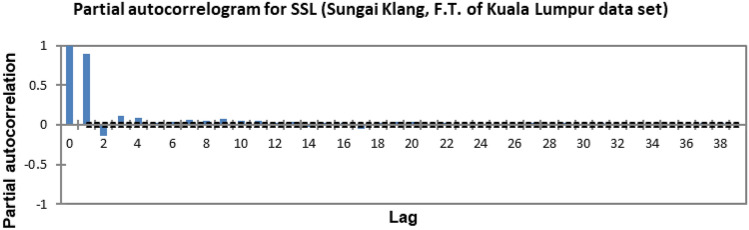
Partial autocorrelogram for SSL (Sungai Klang, Federal Territor y of Kuala Lumpur data set).

In the present study, the time-series forecasting problem of predicting SSL is re-framed into a supervised learning problem by organising the data sets into a sliding window. This step is performed to enable the application of SVM and ANN for time-series forecasting, as they are traditionally not time-series forecasting algorithms. Before organising the data sets into a sliding window, a partial autocorrelation function (PACF) analysis was performed on all SSL data to determine the lagged SSL data that are most correlated to the current-day SSL data. Based on the PACF analyses in Figs. 6, 7, 8, 9, 10, 11, 12, 13, 14, 15, 16, for most data sets, the 1-day lagged SSL [SSL(t-1)], 2-day lagged SSL [SSL(t-2)], and 3-day lagged SSL [SSL(t-3)] are shown to have a significant correlation with the current-day SSL [SSL(t)].

Next, Pearson’s correlation coefficient is employed to analyse the correlation between current-day SF data [SF(t)] and SSL(t). Pearson’s correlation coefficient, denoted by ${r}_{xy},$ is defined by:

9 $${r}_{xy}=\frac{\sum_{i=1}^{n}\left({x}_{i}-\overline{x }\right)\left({y}_{i}-\overline{y }\right)}{\sqrt{\sum_{i=1}^{n}{\left({x}_{i}-\overline{x }\right)}^{2}}\sqrt{\sum_{i=1}^{n}{\left({y}_{i}-\overline{y }\right)}^{2}}}$$

where $\overline{x }$,$\overline{y }$ are respective data means; ${x}_{i},{y}_{i}$ are individual respective data points; and $n$ is the sample size.

The lagged SSL and SF data are then studied using Pearson’s correlation coefficient to understand the predictive powers of these data as input parameters on SSL(t). Pearson’s correlation coefficient matrix for all the data sets is as shown in Table 8. It can be seen that there is indeed significant correlation between SSL(t-1), SSL(t-2), SSL(t-3), SF(t), SF(t-1), SF(t-2), SF(t-3) and SSL(t) in almost all of the data sets.

**Table 8:** Pearson’s correlation coefficient matrix for data sets of each selected river.

Pearson's correlation coefficient matrix based on Sungai Johor, Johor data set								
Variables	SF(t-3)	SF(t-2)	SF(t-1)	SF(t)	SSL(t-3)	SSL(t-2)	SSL(t-1)	SSL(t)
SF(t-3)	**1**	0.957	0.863	0.759	0.827	0.792	0.712	0.623
SF(t-2)	0.957	**1**	0.957	0.863	0.794	0.827	0.792	0.712
SF(t-1)	0.863	0.957	**1**	0.957	0.716	0.794	0.827	0.792
SF(t)	0.759	0.863	0.957	**1**	0.628	0.716	0.794	0.827
SSL(t-3)	0.827	0.794	0.716	0.628	**1**	0.964	0.883	0.789
SSL(t-2)	0.792	0.827	0.794	0.716	0.964	**1**	0.964	0.883
SSL(t-1)	0.712	0.792	0.827	0.794	0.883	0.964	**1**	0.964
SSL(t)	0.623	0.712	0.792	0.827	0.789	0.883	0.964	**1**
**Pearson's correlation coefficient matrix based on Sungai Muda, Kedah data set**								
Variables	SF(t-3)	SF(t-2)	SF(t-1)	SF(t)	SSL(t-3)	SSL(t-2)	SSL(t-1)	SSL(t)
SF(t-3)	**1**	0.926	0.814	0.737	0.483	0.451	0.403	0.370
SF(t-2)	0.926	**1**	0.926	0.814	0.452	0.483	0.451	0.403
SF(t-1)	0.814	0.926	**1**	0.926	0.408	0.452	0.483	0.451
SF(t)	0.737	0.814	0.926	**1**	0.379	0.408	0.452	0.483
SSL(t-3)	0.483	0.452	0.408	0.379	**1**	0.969	0.933	0.913
SSL(t-2)	0.451	0.483	0.452	0.408	0.969	**1**	0.969	0.933
SSL(t-1)	0.403	0.451	0.483	0.452	0.933	0.969	**1**	0.969
SSL(t)	0.370	0.403	0.451	0.483	0.913	0.933	0.969	**1**
**Pearson's correlation coefficient matrix based on Sungai Kelantan, Kelantan data set**								
Variables	SF(t-3)	SF(t-2)	SF(t-1)	SF(t)	SSL(t-3)	SSL(t-2)	SSL(t-1)	SSL(t)
SF(t-3)	**1**	0.904	0.737	0.613	0.454	0.408	0.335	0.283
SF(t-2)	0.904	**1**	0.904	0.737	0.409	0.454	0.408	0.335
SF(t-1)	0.737	0.904	**1**	0.904	0.333	0.409	0.454	0.408
SF(t)	0.613	0.737	0.904	**1**	0.280	0.333	0.409	0.454
SSL(t-3)	0.454	0.409	0.333	0.280	**1**	0.934	0.840	0.776
SSL(t-2)	0.408	0.454	0.409	0.333	0.934	**1**	0.934	0.840
SSL(t-1)	0.335	0.408	0.454	0.409	0.840	0.934	**1**	0.934
SSL(t)	0.283	0.335	0.408	0.454	0.776	0.840	0.934	**1**
**Pearson's correlation coefficient matrix based on Sungai Melaka, Melaka data set**								
Variables	SF(t-3)	SF(t-2)	SF(t-1)	SF(t)	SSL(t-3)	SSL(t-2)	SSL(t-1)	SSL(t)
SF(t-3)	**1**	0.794	0.610	0.528	0.594	0.425	0.293	0.240
SF(t-2)	0.794	**1**	0.794	0.610	0.433	0.594	0.425	0.293
SF(t-1)	0.610	0.794	**1**	0.794	0.313	0.433	0.594	0.425
SF(t)	0.528	0.610	0.794	**1**	0.265	0.313	0.433	0.594
SSL(t-3)	0.594	0.433	0.313	0.265	**1**	0.709	0.510	0.427
SSL(t-2)	0.425	0.594	0.433	0.313	0.709	**1**	0.709	0.510
SSL(t-1)	0.293	0.425	0.594	0.433	0.510	0.709	**1**	0.709
SSL(t)	0.240	0.293	0.425	0.594	0.427	0.510	0.709	**1**
**Pearson's correlation coefficient matrix based on Sungai Kepis, Negeri Sembilan data set**								
Variables	SF(t-3)	SF(t-2)	SF(t-1)	SF(t)	SSL(t-3)	SSL(t-2)	SSL(t-1)	SSL(t)
SF(t-3)	**1**	0.387	0.222	0.115	0.652	0.090	0.035	0.009
SF(t-2)	0.387	**1**	0.387	0.222	0.145	0.652	0.090	0.035
SF(t-1)	0.222	0.387	**1**	0.387	0.056	0.145	0.652	0.090
SF(t)	0.115	0.222	0.387	**1**	0.027	0.056	0.145	0.652
SSL(t-3)	0.652	0.145	0.056	0.027	**1**	0.036	0.014	0.005
SSL(t-2)	0.090	0.652	0.145	0.056	0.036	**1**	0.036	0.014
SSL(t-1)	0.035	0.090	0.652	0.145	0.014	0.036	**1**	0.036
SSL(t)	0.009	0.035	0.090	0.652	0.005	0.014	0.036	**1**
**Pearson's correlation coefficient matrix based on Sungai Pahang, Pahang data set**								
Variables	SF(t-3)	SF(t-2)	SF(t-1)	SF(t)	SSL(t-3)	SSL(t-2)	SSL(t-1)	SSL(t)
SF(t-3)	**1**	0.974	0.919	0.858	0.588	0.583	0.556	0.520
SF(t-2)	0.974	**1**	0.974	0.919	0.568	0.588	0.583	0.556
SF(t-1)	0.919	0.974	**1**	0.974	0.535	0.568	0.588	0.583
SF(t)	0.858	0.919	0.974	**1**	0.498	0.535	0.568	0.588
SSL(t-3)	0.588	0.568	0.535	0.498	**1**	0.980	0.931	0.865
SSL(t-2)	0.583	0.588	0.568	0.535	0.980	**1**	0.980	0.931
SSL(t-1)	0.556	0.583	0.588	0.568	0.931	0.980	**1**	0.980
SSL(t)	0.520	0.556	0.583	0.588	0.865	0.931	0.980	**1**
**Pearson's correlation coefficient matrix based on Sungai Perak, Perak data set**								
Variables	SF(t-3)	SF(t-2)	SF(t-1)	SF(t)	SSL(t-3)	SSL(t-2)	SSL(t-1)	SSL(t)
SF(t-3)	**1**	0.947	0.906	0.880	0.383	0.330	0.293	0.270
SF(t-2)	0.947	**1**	0.947	0.906	0.330	0.383	0.330	0.293
SF(t-1)	0.906	0.947	**1**	0.947	0.291	0.329	0.383	0.330
SF(t)	0.880	0.906	0.947	**1**	0.264	0.291	0.329	0.383
SSL(t-3)	0.383	0.330	0.291	0.264	**1**	0.882	0.808	0.755
SSL(t-2)	0.330	0.383	0.329	0.291	0.882	**1**	0.882	0.808
SSL(t-1)	0.293	0.330	0.383	0.329	0.808	0.882	**1**	0.882
SSL(t)	0.270	0.293	0.330	0.383	0.755	0.808	0.882	**1**
**Pearson's correlation coefficient matrix based on Sungai Perlis, Perlis data set**								
Variables	SF(t-3)	SF(t-2)	SF(t-1)	SF(t)	SSL(t-3)	SSL(t-2)	SSL(t-1)	SSL(t)
SF(t-3)	**1**	0.835	0.684	0.607	0.862	0.708	0.567	0.496
SF(t-2)	0.835	**1**	0.834	0.684	0.714	0.862	0.708	0.567
SF(t-1)	0.684	0.834	**1**	0.834	0.576	0.714	0.862	0.708
SF(t)	0.607	0.684	0.834	**1**	0.511	0.576	0.714	0.862
SSL(t-3)	0.862	0.714	0.576	0.511	**1**	0.785	0.600	0.525
SSL(t-2)	0.708	0.862	0.714	0.576	0.785	**1**	0.785	0.600
SSL(t-1)	0.567	0.708	0.862	0.714	0.600	0.785	**1**	0.785
SSL(t)	0.496	0.567	0.708	0.862	0.525	0.600	0.785	**1**
**Pearson's correlation coefficient matrix based on Sungai Selangor, Selangor data set**								
Variables	SF(t-3)	SF(t-2)	SF(t-1)	SF(t)	SSL(t-3)	SSL(t-2)	SSL(t-1)	SSL(t)
SF(t-3)	**1**	0.944	0.860	0.793	0.782	0.734	0.662	0.605
SF(t-2)	0.944	**1**	0.944	0.860	0.732	0.782	0.734	0.662
SF(t-1)	0.860	0.944	**1**	0.944	0.657	0.732	0.782	0.734
SF(t)	0.793	0.860	0.944	**1**	0.599	0.657	0.732	0.782
SSL(t-3)	0.782	0.732	0.657	0.599	**1**	0.939	0.851	0.784
SSL(t-2)	0.734	0.782	0.732	0.657	0.939	**1**	0.939	0.851
SSL(t-1)	0.662	0.734	0.782	0.732	0.851	0.939	**1**	0.939
SSL(t)	0.605	0.662	0.734	0.782	0.784	0.851	0.939	**1**
**Pearson's correlation coefficient matrix based on Sungai Dungun, Terengganu data set**								
Variables	SF(t-3)	SF(t-2)	SF(t-1)	SF(t)	SSL(t-3)	SSL(t-2)	SSL(t-1)	SSL(t)
SF(t-3)	**1**	0.929	0.810	0.701	0.556	0.534	0.457	0.390
SF(t-2)	0.929	**1**	0.929	0.810	0.484	0.556	0.534	0.457
SF(t-1)	0.810	0.929	**1**	0.929	0.410	0.484	0.556	0.534
SF(t)	0.701	0.810	0.929	**1**	0.351	0.410	0.484	0.556
SSL(t-3)	0.556	0.484	0.410	0.351	**1**	0.897	0.776	0.688
SSL(t-2)	0.534	0.556	0.484	0.410	0.897	**1**	0.897	0.776
SSL(t-1)	0.457	0.534	0.556	0.484	0.776	0.897	**1**	0.897
SSL(t)	0.390	0.457	0.534	0.556	0.688	0.776	0.897	**1**
**Pearson's correlation coefficient matrix based on Sungai Klang, F.T. of Kuala Lumpur data set**								
Variables	SF(t-3)	SF(t-2)	SF(t-1)	SF(t)	SSL(t-3)	SSL(t-2)	SSL(t-1)	SSL(t)
SF(t-3)	**1**	0.489	0.299	0.299	0.989	0.498	0.308	0.311
SF(t-2)	0.489	**1**	0.489	0.299	0.487	0.989	0.498	0.307
SF(t-1)	0.299	0.489	**1**	0.489	0.297	0.488	0.989	0.497
SF(t)	0.299	0.299	0.489	**1**	0.291	0.297	0.487	0.989
SSL(t-3)	0.989	0.487	0.297	0.291	**1**	0.514	0.322	0.317
SSL(t-2)	0.498	0.989	0.488	0.297	0.514	**1**	0.515	0.322
SSL(t-1)	0.308	0.498	0.989	0.487	0.322	0.515	**1**	0.514
SSL(t)	0.311	0.307	0.497	0.989	0.317	0.322	0.514	**1**

Based on the findings from the PACF and Pearson’s correlation coefficient analyses, the SSL(t-1), SSL(t-2), SSL(t-3), SF(t), SF(t-1), SF(t-2), SF(t-3) data are determined to have significant predictive powers over SSL(t), hence are selected as input parameters for the present study. Therefore, in addition to the Date, SSL(t), and SF(t) columns described in Sect. 2.4.1, columns for SSL(t-1), SSL(t-2), SSL(t-3), SF(t-1), SF(t-2), SF(t-3) are also added into the .csv files for each respective river.

Based on these selected input parameters, several input scenarios are formed to train the ML models for SSL prediction. The training of the models using different input scenarios is also performed to test the sensitivity of the models to different input combinations, similar to existing studies^2,8,9,23,24,26,35^. The designed input parameter scenarios are shown in Table 9. With 4 input parameter scenarios, 3 ML algorithms, and 11 data sets, a total of 132 models were run and evaluated in total.

**Table 9:** Input parameter scenarios designed for the present study.

Output parameter	Input parameter scenario	Input parameter(s)	Description
SSL(t)	1	SSL(t-1)	When SSL data of previous day is available
	2	SSL(t-1) + SSL(t-2) + SSL (t-3)	When SSL data of previous 3 days is available
	3	SSL(t-1) + SF(t) + SF (t-1)	When SSL data of previous day and SF data of current day and previous day is available
	4	SSL(t-1) + SSL(t-2) + SSL (t-3) + SF(t) + SF(t-1) + SF(t-2) + SF(t-3)	When SSL data and SF data of previous 3 days is available

### Performance measures

Four performance measures are selected to evaluate the models’ performances, namely the mean absolute error (MAE), root mean squared error (RMSE), coefficient of determination (R^2^), and ranking mean (RM). MAE, RMSE, and R^2^ have been commonly used in SSL prediction studies^2,8–10,19,21,22,24,37,38,69^, while RM was used by Ahmed et al.^70^ as a method to rank overall model performance.

#### Mean absolute error (MAE)

The MAE quantifies the average absolute difference between predicted values and actual values. Therefore, a lower MAE is desired. In the present study, the MAE is measured in units of ton/day. The MAE is defined by:

10 $$MAE=\frac{1}{n}\bullet \left[\sum_{i=1}^{n}\left|{y}_{i}-\widehat{{y}_{i}}\right|\right]$$

where ${y}_{i}$ is the real value, $\widehat{{y}_{i}}$ is the predicted value, and $n$ is the sample size.

#### Root mean squared error (RMSE)

The RMSE is a good indicator of large errors as it places a relatively high weight to large errors. A lower RMSE is generally desired. The present study measures the RMSE in units of ton/day. The equation for computing the RMSE is as follows:

11 $$RMSE=\sqrt{\frac{1}{n}\bullet \left[\sum_{i=1}^{n}{\left({y}_{i}-\widehat{{y}_{i}}\right)}^{2}\right]}$$

where ${y}_{i}$ is the real value, $\widehat{{y}_{i}}$ is the predicted value, and $n$ is the sample size.

#### *Coefficient of determination (R*^*2*^*)*

The R^2^ essentially calculates the correlation between real values and predicted values. R^2^ scores may lie between − 1 and 1, with a value closer to 1 signalling a higher correlation between real values and predicted values. R^2^ scores are unitless. To calculate R^2^, the following equation is used:

12 $${R}^{2}=1-\left[\frac{\sum_{i=1}^{n}{\left({y}_{i}-\widehat{{y}_{i}}\right)}^{2}}{\sum_{i=1}^{n}{\left({y}_{i}-\overline{{y }_{i}}\right)}^{2}}\right]$$

where ${y}_{i}$ is real value, $\widehat{{y}_{i}}$ is predicted value, $\overline{{y }_{i}}$ is the mean of ${y}_{i}$, and $n$ is sample size.

#### Ranking mean (RM)

Each model is first ranked based on the scores of the selected performance measures in the present study, which are MAE, RMSE, and R^2^. Then, the RM of each model is calculated by averaging the ranks based on the scores of the three performance measures, MAE, RMSE, and R^2^. The higher the RM, the better the overall performance of a model. The RM is represented by the formula:

13 $$RM=\frac{1}{n}\sum_{i=1}^{n}{rank}_{i}$$

where $n$ is the number of performance analysis measures used, which is 3.

## Results and discussion

This section presents and discusses the performances of the developed models for SSL prediction. Comparisons and analyses are then made based on the model performances.

### Performance of models based on the Sungai Johor, Johor data set

Model ANN3, based on the ANN algorithm and input parameter scenario 3, produced the best overall performance in predicting the SSL for the Sungai Johor, Johor data set.

**Figure 17:**
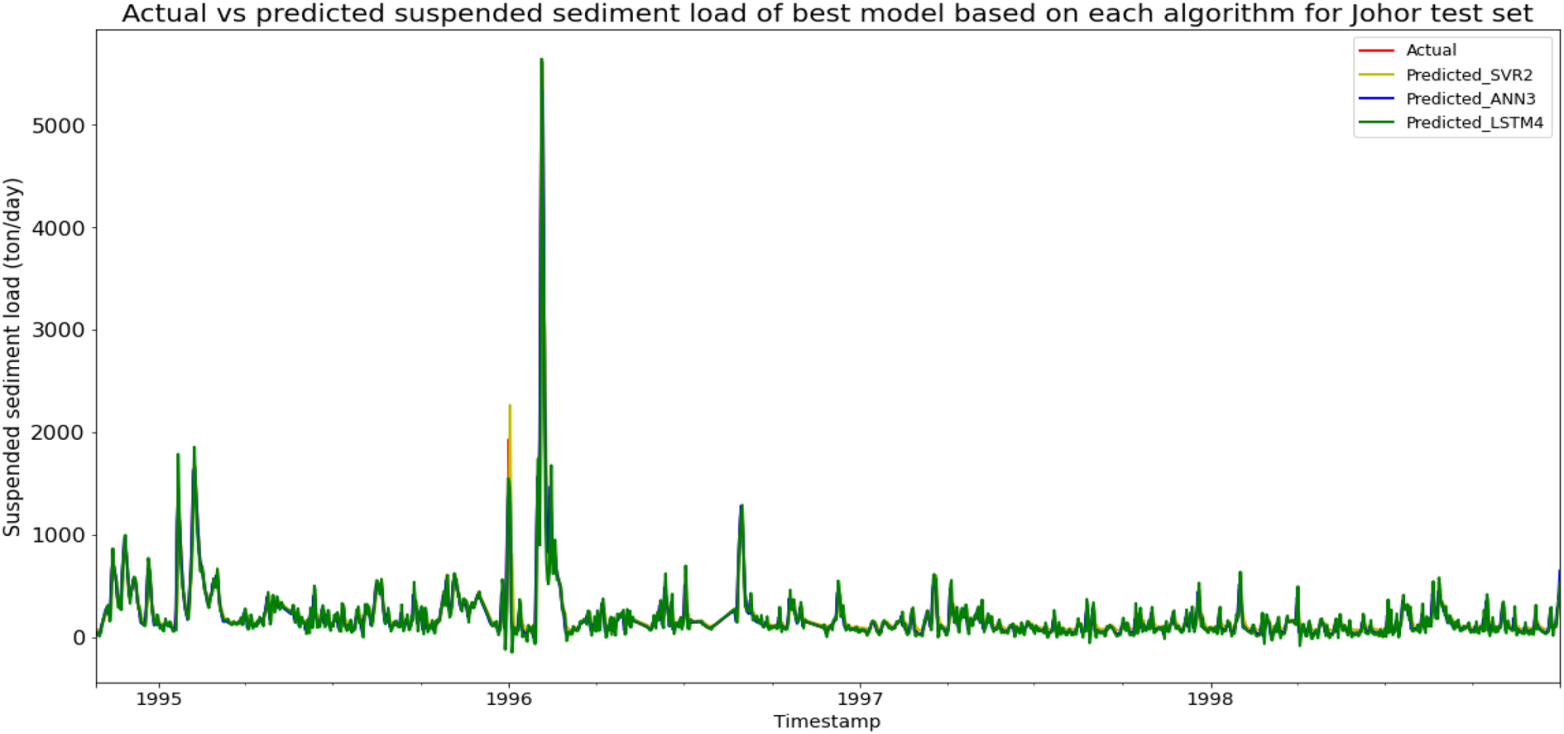
Actual vs predicted SSL of best models based on each algorithm for Sungai Johor test set.

ANN3 achieved the best MAE, RMSE, and R^2^ with scores of 13.7489 ton/day, 28.4590 ton/day, and 0.9918 respectively, hence giving it the highest RM of 1.00. The best SVR model was SVR2 (RM = 6.33), while the best LSTM model was LSTM4 (RM = 5.67). The models’ performance scores and actual vs predicted SSL of best models from each algorithm for the Sungai Johor test set is shown in Table 10 and Fig. 17 respectively.

**Table 10:** Models’ performance scores based on Sungai Johor test set.

Model	MAE	RMSE	R2	Rank (MAE)	Rank (RMSE)	Rank (R^2^)	RM
SVR1	47.1793	118.7276	0.8568	10	8	8	8.67
SVR2	41.6048	111.2320	0.8743	7	6	6	6.33
SVR3	24.9926	122.3341	0.8479	3	9	9	7.00
SVR4	29.1360	142.1895	0.7945	4	10	10	8.00
ANN1	39.7224	113.2527	0.8697	6	7	7	6.67
ANN2	29.7550	80.2473	0.9346	5	3	3	3.67
ANN3	13.7489	28.4590	0.9918	1	1	1	1.00
ANN4	20.6536	37.1175	0.9860	2	2	2	2.00
LSTM1	59.2260	166.5459	0.7190	11	11	11	11.00
LSTM2	60.1567	169.0806	0.7104	12	12	12	12.00
LSTM3	42.6391	90.5516	0.9169	8	5	5	6.00
LSTM4	43.5163	88.5755	0.9205	9	4	4	5.67

### Performance of models based on the Sungai Muda, Kedah data set

**Figure 18:**
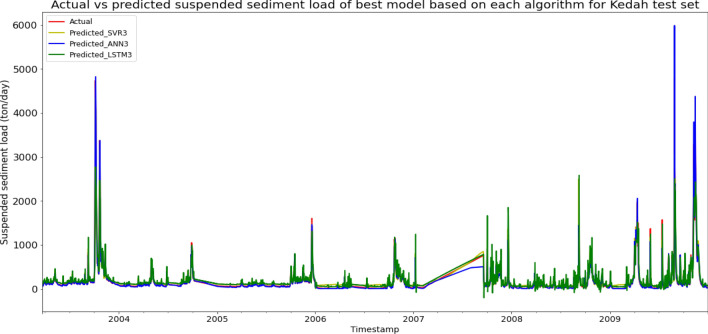
Actual vs predicted SSL of best models based on each algorithm for Sungai Muda test set.

Model ANN3, based on the ANN algorithm and input parameter scenario 3, produced the best overall performance in predicting the SSL for the Sungai Muda, Kedah data set. ANN3 achieved the best MAE, RMSE, and R^2^ with scores of 28.3826 ton/day, 76.4909 ton/day, and 0.9548 respectively, hence giving it the highest RM of 1.00. The best SVR model was SVR3 (RM = 3.00), while the best LSTM model was LSTM3 (RM = 8.67). The models’ performance scores and actual vs predicted SSL of best models from each algorithm for the Sungai Muda test set is shown in Table 11 and Fig. 18 respectively.

**Table 11:** Models’ performance scores based on Sungai Muda test set.

Model	MAE	RMSE	R2	Rank (MAE)	Rank (RMSE)	Rank (R^2^)	RM
SVR1	97.2995	173.7792	0.7669	12	8	8	9.33
SVR2	95.5567	166.7496	0.7854	11	7	7	8.33
SVR3	47.7671	83.1980	0.9466	5	2	2	3.00
SVR4	40.2587	92.3426	0.9342	3	4	4	3.67
ANN1	50.9026	161.8786	0.7977	6	6	6	6.00
ANN2	47.5865	153.4338	0.8183	4	5	5	4.67
ANN3	28.3826	76.4909	0.9548	1	1	1	1.00
ANN4	33.0079	87.8177	0.9405	2	3	3	2.67
LSTM1	82.8842	245.7265	0.5351	10	11	11	10.67
LSTM2	75.4008	245.9534	0.5342	7	12	12	10.33
LSTM3	81.0806	182.1500	0.7445	8	9	9	8.67
LSTM4	81.7226	183.3309	0.7412	9	10	10	9.67

### Performance of models based on the Sungai Kelantan, Kelantan data set

**Figure 19:**
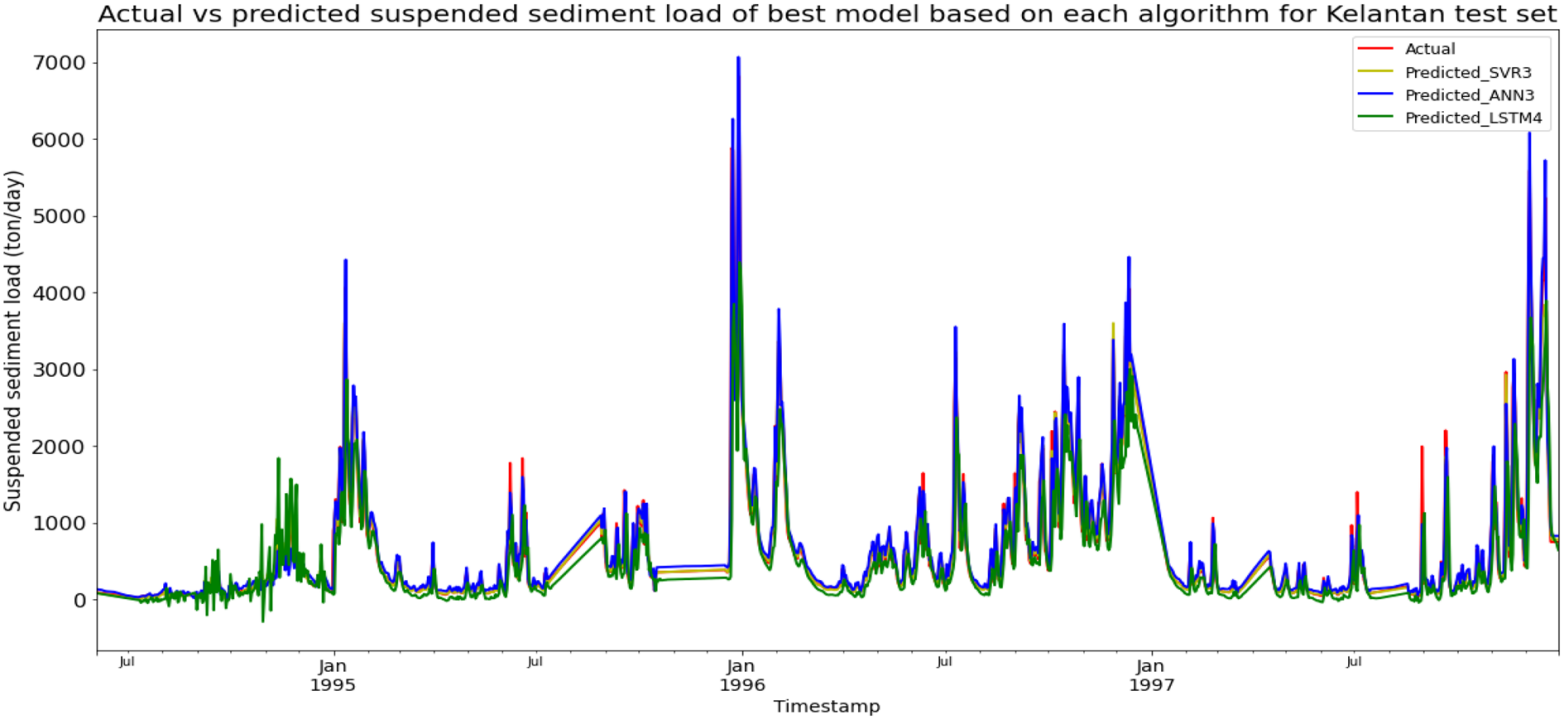
Actual vs predicted SSL of best models based on each algorithm for Sungai Kelantan test set.

Model ANN3, based on the ANN algorithm and input parameter scenario 3, produced the best overall performance in predicting the SSL for the Sungai Kelantan, Kelantan data set. ANN3 achieved the best RMSE and R^2^ with scores of 126.0058 ton/day and 0.9761 respectively, hence giving it the highest RM of 1.67. SVR3 obtained the best MAE with a score of 53.7383 ton/day. The best SVR model was SVR3 (RM = 2.33), while the best LSTM model was LSTM4 (RM = 9.00). The models’ performance scores and actual vs predicted SSL of best models from each algorithm for the Sungai Kelantan test set is shown in Table 12 and Fig. 19 respectively.

**Table 12:** Models’ performance scores based on Sungai Kelantan test set.

Model	MAE	RMSE	R2	Rank (MAE)	Rank (RMSE)	Rank (R^2^)	RM
SVR1	151.4002	340.0729	0.8259	7	8	8	7.67
SVR2	147.4009	337.1593	0.8289	6	7	7	6.67
SVR3	53.7383	169.0265	0.9570	1	3	3	2.33
SVR4	85.6392	272.5014	0.8882	4	4	4	4.00
ANN1	153.4962	334.9441	0.8312	8	6	6	6.67
ANN2	143.7879	317.0991	0.8487	5	5	5	5.00
ANN3	81.9359	126.0058	0.9761	3	1	1	1.67
ANN4	68.8114	144.2213	0.9687	2	2	2	2.00
LSTM1	244.7703	504.4452	0.6182	12	11	11	11.33
LSTM2	235.3755	508.0856	0.6127	10	12	12	11.33
LSTM3	238.9999	438.4423	0.7116	11	10	10	10.33
LSTM4	214.0424	424.9830	0.7290	9	9	9	9.00

### Performance of models based on the Sungai Melaka, Melaka data set

**Figure 20:**
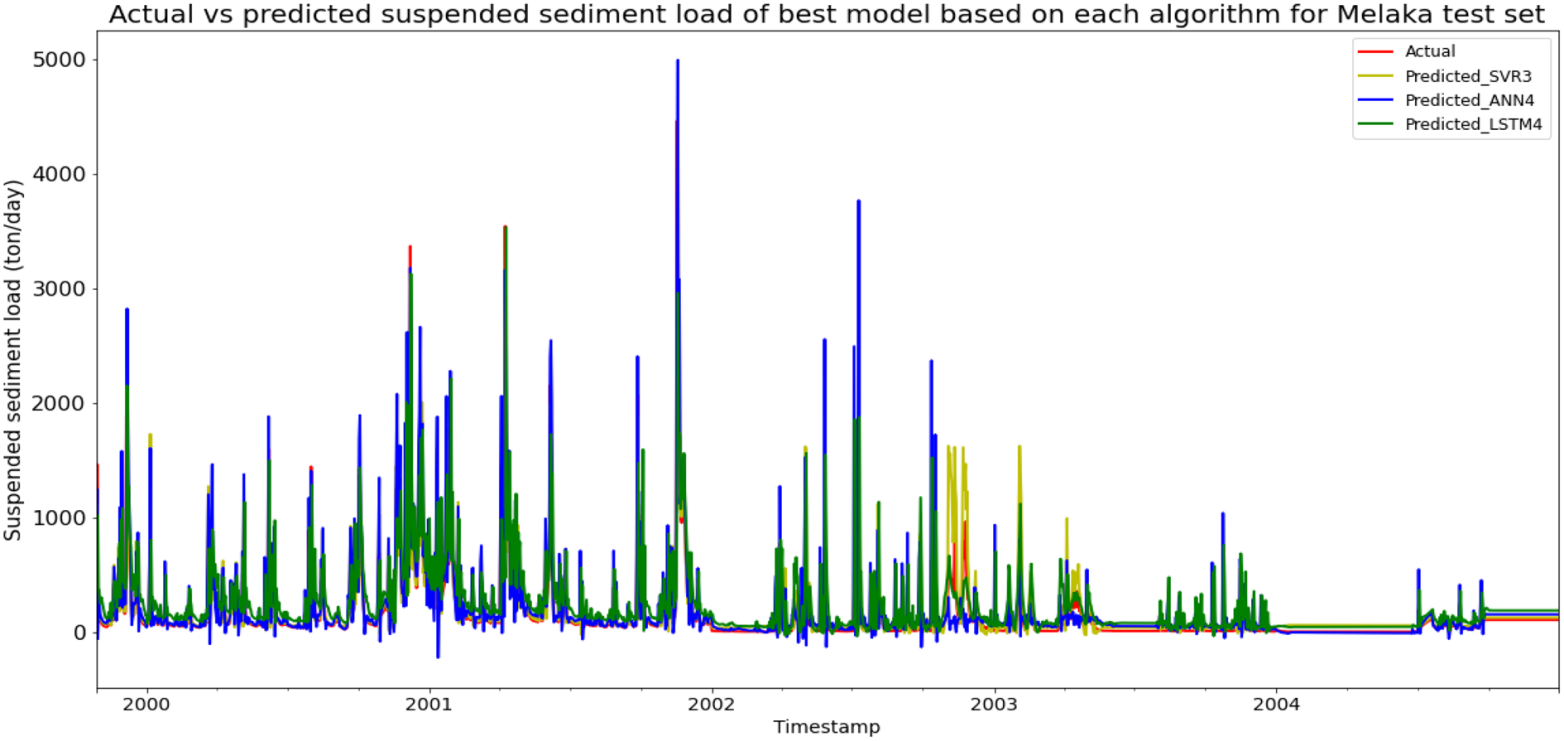
Actual vs predicted SSL of best models based on each algorithm for Sungai Melaka test set.

Model SVR3, based on the SVR algorithm and input parameter scenario 3, produced the best overall performance in predicting the SSL for the Sungai Melaka, Melaka data set. SVR3 achieved the best MAE, RMSE, and R^2^ with scores of 62.3282 ton/day, 149.6537 ton/day, and 0.7787 respectively, hence giving it the highest RM of 1.00. The best ANN model was ANN4 (RM = 3.00), while the best LSTM model was LSTM3 (RM = 9.00). The models’ performance scores and actual vs predicted SSL of best models from each algorithm for Sungai Melaka test set is shown in Table 13 and Fig. 20 respectively.

**Table 13:** Models’ performance scores based on Sungai Melaka test set.

Model	MAE	RMSE	R2	Rank (MAE)	Rank (RMSE)	Rank (R^2^)	RM
SVR1	90.0460	231.2991	0.4714	6	6	6	6.00
SVR2	88.6399	229.7522	0.4785	5	5	5	5.00
SVR3	62.3282	149.6537	0.7787	1	1	1	1.00
SVR4	66.5554	159.9368	0.7473	2	2	2	2.00
ANN1	130.3886	235.9549	0.4500	8	8	8	8.00
ANN2	129.5363	234.3656	0.4573	7	7	7	7.00
ANN3	86.6945	187.6765	0.6520	4	4	4	4.00
ANN4	82.3402	181.5299	0.6744	3	3	3	3.00
LSTM1	215.4332	302.4288	0.0996	12	12	12	12.00
LSTM2	197.4509	299.9267	0.1144	11	11	11	11.00
LSTM3	165.1691	296.4999	0.1346	10	10	10	10.00
LSTM4	141.9856	262.8415	0.3199	9	9	9	9.00

### Performance of models based on the Sungai Kepis, Negeri Sembilan data set

**Figure 21:**
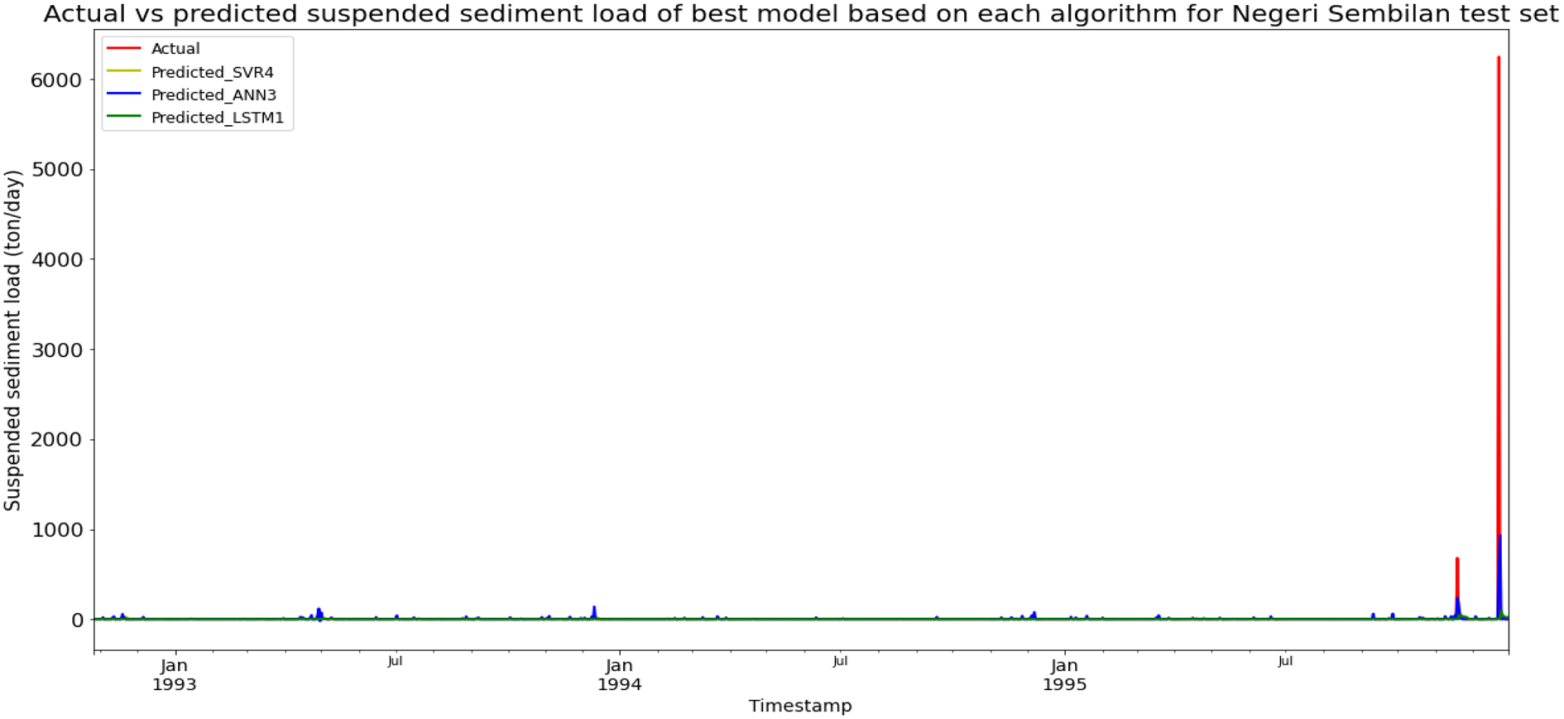
Actual vs predicted SSL of best models based on each algorithm for Sungai Kepis test set.

Model ANN3, based on the ANN algorithm and input parameter scenario 3, produced the best overall performance in predicting the SSL for the Sungai Kepis, Negeri Sembilan data set. ANN3 achieved the best RMSE and R^2^ with scores of 170.9490 ton/day and 0.1340 respectively, hence giving it the highest RM of 2.67. LSTM1 obtained the best MAE with a score of 7.0221 ton/day. The best SVR model was SVR4 (RM = 3.67), while the best LSTM model was LSTM1 (RM = 5.00). The models’ performance scores and actual vs predicted SSL of best models from each algorithm for the Sungai Kepis test set is shown in Table 14 and Fig. 21 respectively.

**Table 14:** Models’ performance scores based on Sungai Kepis test set.

Model	MAE	RMSE	R2	Rank (MAE)	Rank (RMSE)	Rank (R^2^)	RM
SVR1	8.5567	183.7401	− 0.0004	5	6	6	5.67
SVR2	8.5120	183.7365	− 0.0004	4	5	5	4.67
SVR3	8.7057	182.4586	0.0135	7	3	3	4.33
SVR4	7.8675	183.1118	0.0064	3	4	4	3.67
ANN1	9.0731	198.4062	− 0.1665	8	11	11	10.00
ANN2	10.5838	208.6346	− 0.2899	12	12	12	12.00
ANN3	8.5684	170.9490	0.1340	6	1	1	2.67
ANN4	10.1998	177.9901	0.0612	11	2	2	5.00
LSTM1	7.0221	184.4076	− 0.0017	1	7	7	5.00
LSTM2	7.2435	184.6410	− 0.0042	2	8	8	6.00
LSTM3	9.1092	184.7992	− 0.0059	9	9	9	9.00
LSTM4	9.5115	184.8202	− 0.0061	10	10	10	10.00

### Performance of models based on the Sungai Pahang, Pahang data set

**Figure 22:**
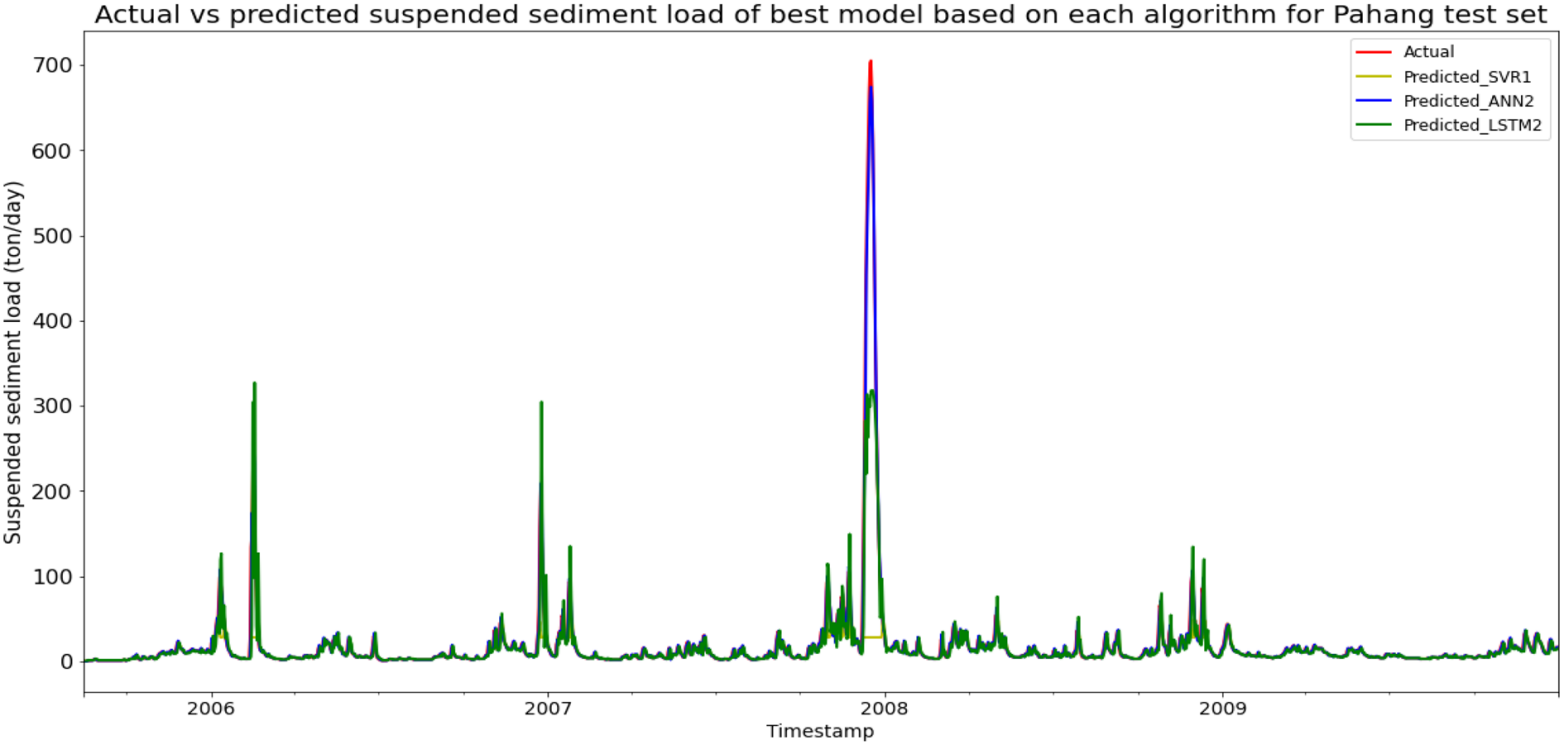
Actual vs predicted SSL of best models based on each algorithm for Sungai Pahang test set.

Model ANN2, based on the ANN algorithm and input parameter scenario 2, produced the best overall performance in predicting the SSL for Sungai Pahang, Pahang data set. ANN2 achieved the best MAE, RMSE, and R^2^ with scores of 2.6228 ton/day, 7.6295 ton/day, and 0.9795 respectively, hence giving it the highest RM of 1.00. The best SVR model was SVR1 (RM = 8.33), while the best LSTM model was LSTM2 (RM = 3.33). The models’ performance scores and actual vs predicted SSL of best models from each algorithm for Sungai Pahang test set is shown in Table 15 and Fig. 22 respectively.

**Table 15:** Models’ performance scores based on Sungai Pahang test set.

Model	MAE	RMSE	R2	Rank (MAE)	Rank (RMSE)	Rank (R^2^)	RM
SVR1	8.6849	50.6481	0.0974	7	9	9	8.33
SVR2	8.5916	50.8337	0.0907	6	12	12	10.00
SVR3	9.0650	50.6613	0.0969	10	10	10	10.00
SVR4	9.0993	50.7911	0.0923	11	11	11	11.00
ANN1	3.7568	12.9546	0.9409	2	2	2	2.00
ANN2	2.6228	7.6295	0.9795	1	1	1	1.00
ANN3	10.7227	44.5757	0.3008	12	7	7	8.67
ANN4	7.5714	33.1561	0.6132	5	5	5	5.00
LSTM1	6.9055	29.2162	0.7008	3	4	4	3.67
LSTM2	6.9775	28.2581	0.7201	4	3	3	3.33
LSTM3	9.0082	43.6166	0.3332	9	6	6	7.00
LSTM4	8.7162	44.8583	0.2947	8	8	8	8.00

### Performance of models based on the Sungai Perak, Perak data set

**Figure 23:**
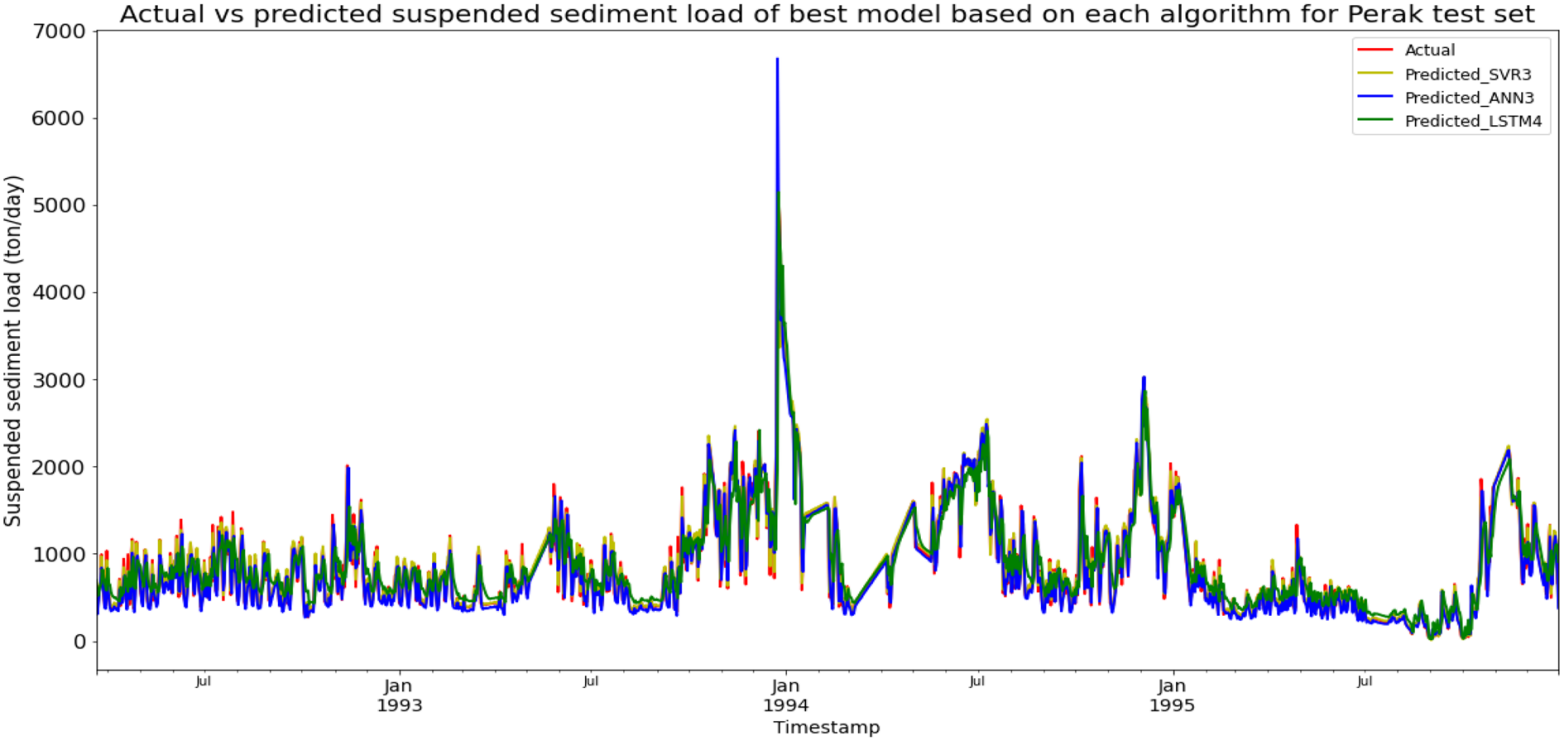
Actual vs predicted SSL of best models based on each algorithm for Sungai Perak test set.

Model SVR3, based on the SVR algorithm and input parameter scenario 3, produced the best overall performance in predicting the SSL for the Sungai Perak, Perak data set. SVR3 achieved the best MAE, RMSE, and R^2^ with scores of 38.0924 ton/day, 82.0057 ton/day, and 0.9817 respectively, hence giving it the highest RM of 1.00. The best ANN model was ANN3 (RM = 3.00), while the best LSTM model was LSTM4 (RM = 7.67). The models’ performance scores and actual vs predicted SSL of best models from each algorithm for the Sungai Perak test set is shown in Table 16 and Fig. 23 respectively.

**Table 16:** Models’ performance scores based on Sungai Perak test set.

Model	MAE	RMSE	R2	Rank (MAE)	Rank (RMSE)	Rank (R^2^)	RM
SVR1	172.2583	263.6394	0.8109	6	6	6	6.00
SVR2	171.0943	262.1442	0.8130	5	5	5	5.00
SVR3	38.0924	82.0057	0.9817	1	1	1	1.00
SVR4	44.7664	103.9075	0.9706	2	2	2	2.00
ANN1	179.9728	268.0674	0.8045	7	8	8	7.67
ANN2	185.1508	275.2471	0.7939	8	10	10	9.33
ANN3	79.1228	126.6130	0.9564	3	3	3	3.00
ANN4	122.5226	177.1150	0.9147	4	4	4	4.00
LSTM1	269.7520	362.6726	0.6433	12	12	12	12.00
LSTM2	260.4905	356.5332	0.6553	11	11	11	11.00
LSTM3	193.1258	269.9014	0.8024	10	9	9	9.33
LSTM4	189.1196	265.6541	0.8086	9	7	7	7.67

### Performance of models based on the Sungai Arau, Perlis data set

**Figure 24:**
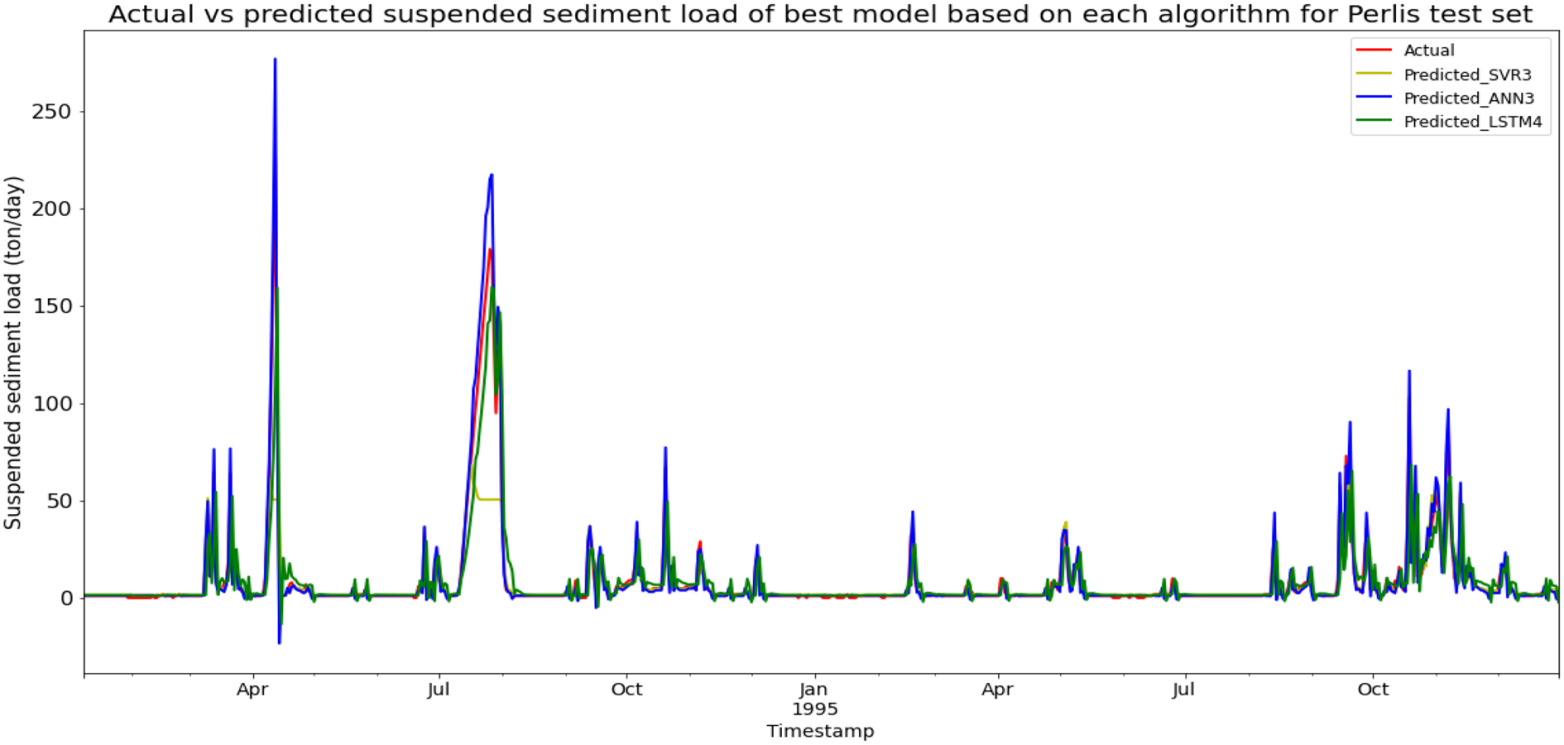
Actual vs predicted SSL of best models based on each algorithm for Sungai Arau test set.

Model ANN3, based on the ANN algorithm and input parameter scenario 3, produced the best overall performance in predicting the SSL for the Sungai Arau, Perlis data set. ANN3 achieved the best MAE, RMSE, and R^2^ with scores of 2.2241 ton/day, 5.3676 ton/day, and 0.9502 respectively, hence giving it the highest RM of 1.00. The best SVR model was SVR3 (RM = 5.67), while the best LSTM model was LSTM4 (RM = 5.00). The models’ performance scores and actual vs predicted SSL of best models from each algorithm for the Sungai Arau test set is shown in Table 17 and Fig. 24 respectively.

**Table 17:** Models’ performance scores based on Sungai Arau test set.

Model	MAE	RMSE	R2	Rank (MAE)	Rank (RMSE)	Rank (R^2^)	RM
SVR1	5.6436	16.2006	0.5461	9	8	8	8.33
SVR2	5.8481	18.4148	0.4136	10	12	12	11.33
SVR3	3.4843	14.6974	0.6264	3	7	7	5.67
SVR4	4.2233	17.5056	0.4700	5	9	9	7.67
ANN1	4.8854	12.5868	0.7260	6	6	6	6.00
ANN2	4.2200	11.6421	0.7656	4	3	3	3.33
ANN3	2.2241	5.3676	0.9502	1	1	1	1.00
ANN4	3.2161	11.1742	0.7841	2	2	2	2.00
LSTM1	7.4557	17.7458	0.4600	12	11	11	11.33
LSTM2	7.1530	17.6884	0.4635	11	10	10	10.33
LSTM3	5.5737	12.5244	0.7310	8	5	5	6.00
LSTM4	5.3228	11.9949	0.7533	7	4	4	5.00

### Performance of models based on the Sungai Selangor, Selangor data set

**Figure 25:**
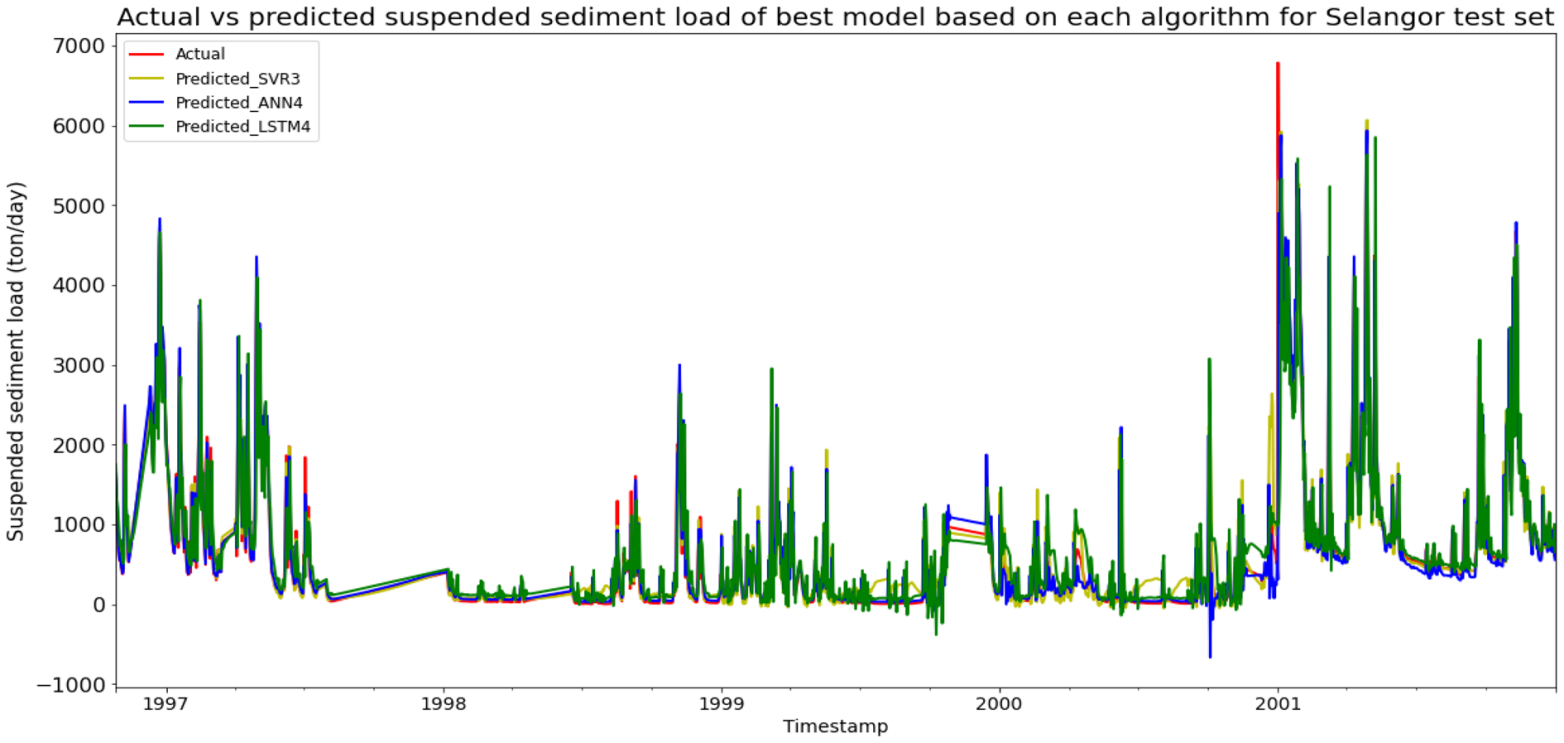
Actual vs predicted SSL of best models based on each algorithm for Sungai Selangor test set.

Model ANN4, based on the ANN algorithm and input parameter scenario 4, produced the best overall performance in predicting the SSL for Sungai Selangor data set. ANN4 achieved the best MAE, RMSE, and R^2^ with scores of 81.7882 ton/day, 209.1255 ton/day, and 0.9425 respectively, hence giving it the highest RM of 1.00. The best SVR model was SVR3 (RM = 3.00), while the best LSTM model was LSTM4 (RM = 9.00). The models’ performance scores and actual vs predicted SSL of best models from each algorithm for Sungai Selangor test set is shown in Table 18 and Fig. 25 respectively.

**Table 18:** Models’ performance scores based on Sungai Selangor test set.

Model	MAE	RMSE	R2	Rank (MAE)	Rank (RMSE)	Rank (R^2^)	RM
SVR1	163.5482	340.4909	0.8476	6	5	5	5.33
SVR2	163.8862	343.5930	0.8448	7	7	7	7.00
SVR3	105.2593	229.1295	0.9310	3	3	3	3.00
SVR4	110.7676	244.8410	0.9212	4	4	4	4.00
ANN1	178.1824	342.0826	0.8461	8	6	6	6.67
ANN2	158.7238	345.9227	0.8427	5	8	8	7.00
ANN3	100.8560	221.7469	0.9353	2	2	2	2.00
ANN4	81.7882	209.1255	0.9425	1	1	1	1.00
LSTM1	281.1844	477.9614	0.6915	12	11	11	11.33
LSTM2	280.3071	478.6574	0.6906	11	12	12	11.67
LSTM3	225.7434	416.8863	0.7653	10	10	10	10.00
LSTM4	215.8317	416.2441	0.7660	9	9	9	9.00

### Performance of models based on the Sungai Dungun, Terengganu data set

**Figure 26:**
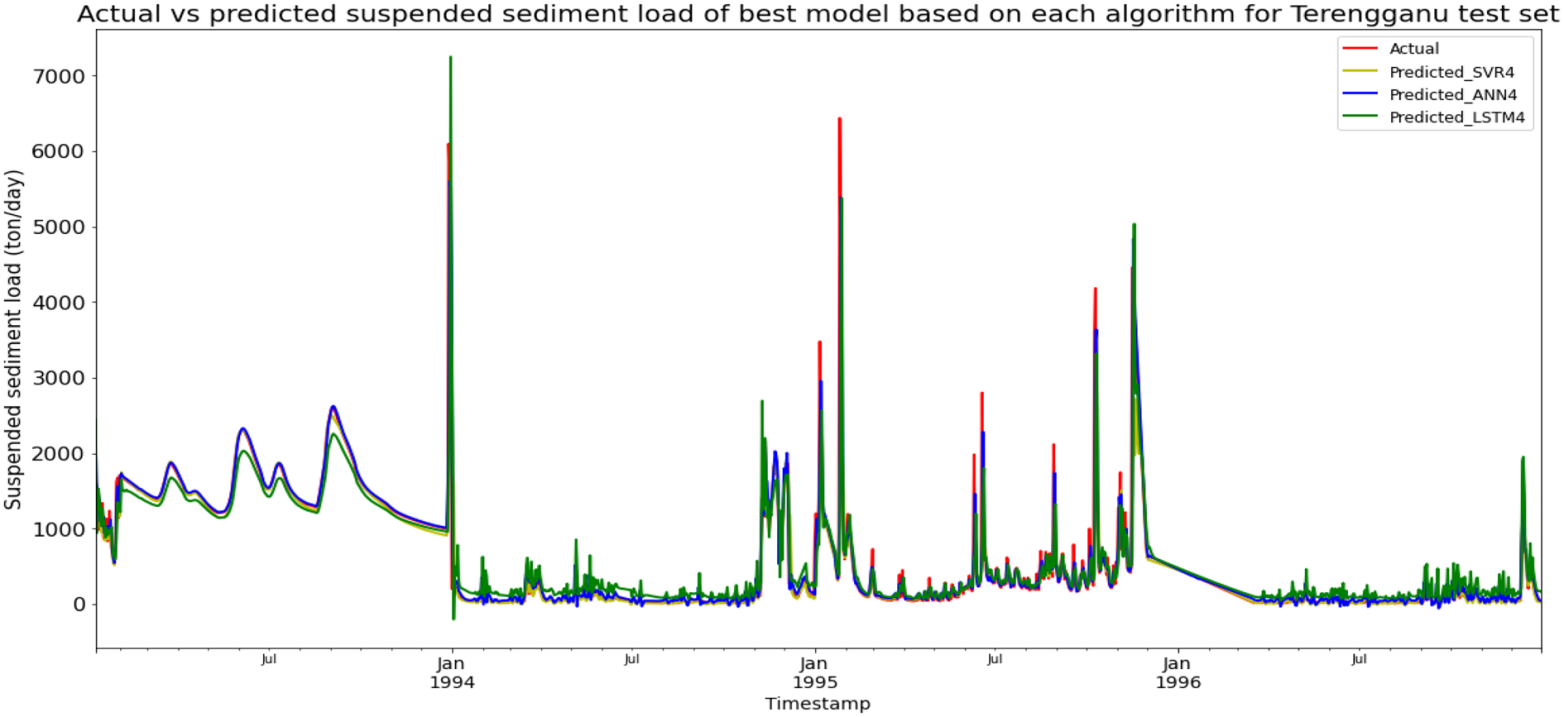
Actual vs predicted SSL of best models based on each algorithm for Sungai Dungun test set.

Model ANN4, based on the ANN algorithm and input parameter scenario 4, produced the best overall performance in predicting the SSL for the Sungai Dungun, Terengganu data set. ANN4 achieved the best RMSE and R^2^ with scores of 287.5243 ton/day and 0.8674 respectively, hence giving it the highest RM of 1.33. ANN3 obtained the best MAE with a score of 68.8483 ton/day. The best SVR model was SVR4 (RM = 3.67), while the best LSTM model was LSTM4 (RM = 8.00). The models’ performance scores and actual vs predicted SSL of best models from each algorithm for the Sungai Dungun test set is shown in Table 19 and Fig. 26 respectively.

**Table 19:** Models’ performance scores based on Sungai Dungun test set.

Model	MAE	RMSE	R2	Rank (MAE)	Rank (RMSE)	Rank (R^2^)	RM
SVR1	124.0464	357.9365	0.7945	8	8	7	7.67
SVR2	119.8292	382.4797	0.7654	7	9	9	8.33
SVR3	111.6157	313.5950	0.8423	6	3	3	4.00
SVR4	79.1380	326.7331	0.8288	3	4	4	3.67
ANN1	101.8140	350.3639	0.8031	4	6	6	5.33
ANN2	107.7054	349.1494	0.8045	5	5	5	5.00
ANN3	68.8483	290.1393	0.8650	1	2	2	1.67
ANN4	75.0303	287.5243	0.8674	2	1	1	1.33
LSTM1	204.0777	455.1370	0.6509	12	12	12	12.00
LSTM2	195.3278	452.3804	0.6551	11	11	11	11.00
LSTM3	186.9991	409.1535	0.7179	10	10	10	10.00
LSTM4	147.6853	356.0388	0.7864	9	7	8	8.00

### Performance of models based on the Sungai Klang, Kuala Lumpur data set

**Figure 27:**
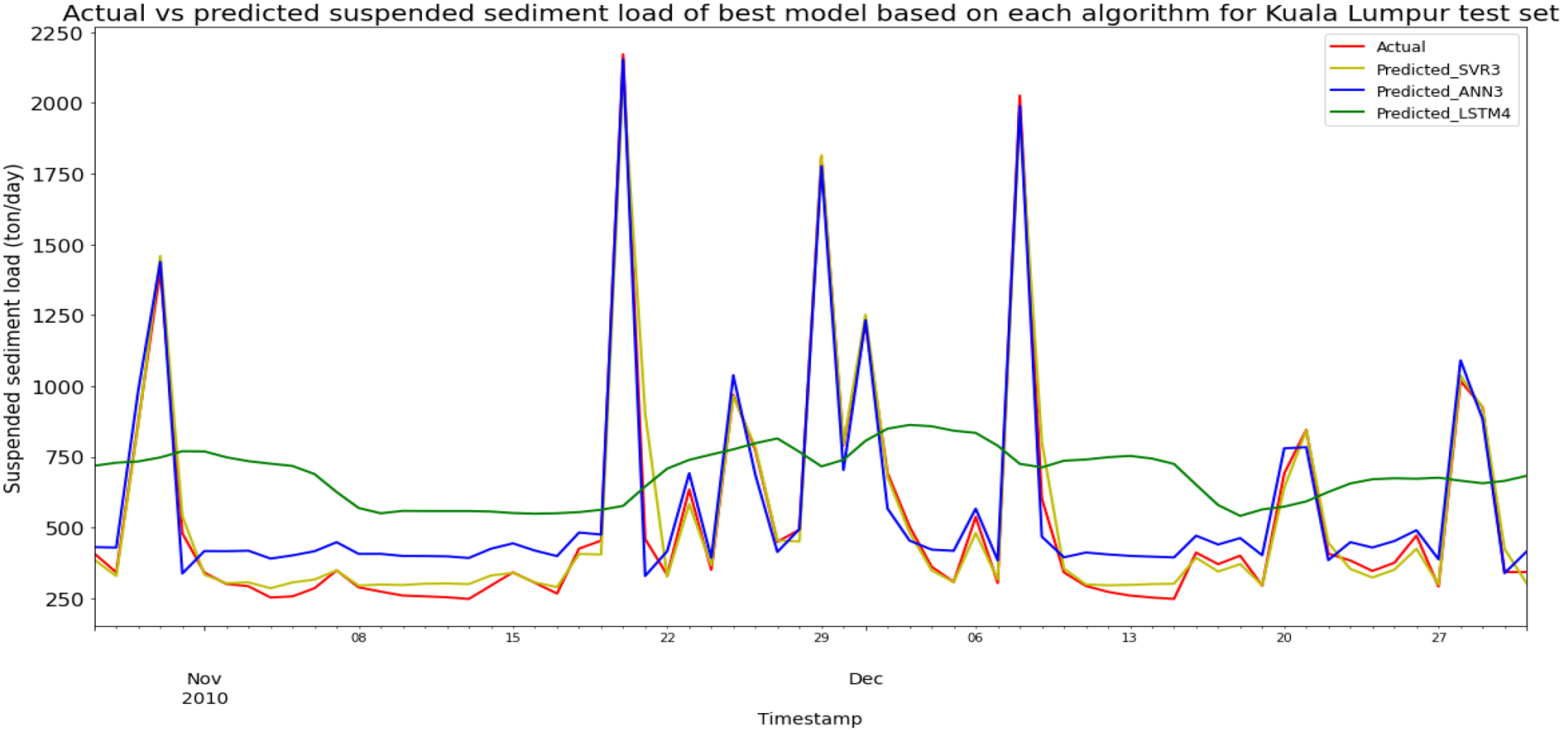
Actual vs predicted SSL of best models based on each algorithm for Sungai Klang test set.

Model SVR3, based on the ANN algorithm and input parameter scenario 3, produced the best overall performance in predicting the SSL for Sungai Klang data set. SVR3 achieved the best MAE, RMSE, and R^2^ with scores 33.8257 ton/day, 65.4953 ton/day, and 0.9721 respectively, hence giving it the highest RM of 1.00. The best ANN model was ANN3 (RM = 2.33), while the best LSTM model was LSTM4 (RM = 7.67). The models’ performance scores and actual vs predicted SSL of best models from each algorithm for Sungai Klang test set is shown in Table 20 and Fig. 27 respectively.

**Table 20:** Models’ performance scores based on Sungai Klang test set.

Model	MAE	RMSE	R2	Rank (MAE)	Rank (RMSE)	Rank (R^2^)	RM
SVR1	234.9908	408.5534	− 0.0845	6	6	6	6.00
SVR2	233.1373	404.5411	− 0.0633	5	5	5	5.00
SVR3	33.8257	65.4953	0.9721	1	1	1	1.00
SVR4	66.6663	116.0418	0.9125	2	3	3	2.67
ANN1	321.1464	467.1223	− 0.4177	7	8	9	8.00
ANN2	354.7978	482.6928	− 0.5138	8	10	11	9.67
ANN3	85.0203	94.9095	0.9415	3	2	2	2.33
ANN4	109.7693	166.4777	0.8199	4	4	4	4.00
LSTM1	469.6151	520.4282	− 0.6467	12	12	12	12.00
LSTM2	431.0026	487.5906	− 0.4454	11	11	10	10.67
LSTM3	422.4213	480.2891	− 0.4025	10	9	8	9.00
LSTM4	356.9486	436.4166	− 0.1580	9	7	7	7.67

### Overall comparison and analysis of model performances

The models’ performances are compared and analysed based on two evaluations, which are the number of times a model produced the best predictive performance for a data set, and the reliability of each model in producing relatively high-accuracy predictions for different data sets. With regards to the number of times a model produced the best predictive performance for a data set, it is found that ANN3 performed the best in 5 out of the 11 tested data sets, which are the Sungai Johor, Sungai Muda, Sungai Kelantan, Sungai Kepis, and Sungai Arau data sets. SVR3 outperformed the other models in 3 of the tested data sets, namely the Sungai Melaka, Sungai Perak, and Sungai Klang data sets. ANN4 produced the best predictive performance in 2 of the tested data sets which are Sungai Selangor and Sungai Dungun, while ANN2 was the best predictive model for 1 data set which is the Sungai Pahang data set. Therefore, ANN3 was the most accurate SSL predictive model for more data sets compared to the other tested models. It is found that the algorithm and the input scenario that produced the best predictive performance for the most data sets are the ANN and input scenario 3 respectively, as both have the best SSL prediction performance for 8 out of 11 data sets. A matrix of most accurate algorithm and input scenario for each data set and the parameters with highest number of best prediction results can be observed in Tables 21 and 22.

**Table 21:** Matrix of most accurate algorithm and input scenario for each data set.

Algorithm	Input scenario 1	Input scenario 2	Input scenario 3	Input scenario 4
**SVR**			Sungai Melaka, Sungai Perak, Sungai Klang	
**ANN**		Sungai Pahang	Sungai Johor, Sungai Muda, Sungai Kelantan, Sungai Kepis, Sungai Arau	Sungai Selangor, Sungai Dungun
**LSTM**

**Table 22:** Parameters with highest number of best prediction results.

Parameter	Description
Algorithm	ANN *(produced best prediction results in 8/11 data sets)*
Input scenario	Input scenario 3 *(produced best prediction results in 8/11 data sets)*
Model	ANN3 *(produced best prediction results in 5/11 data sets)*

**Figure 28:**
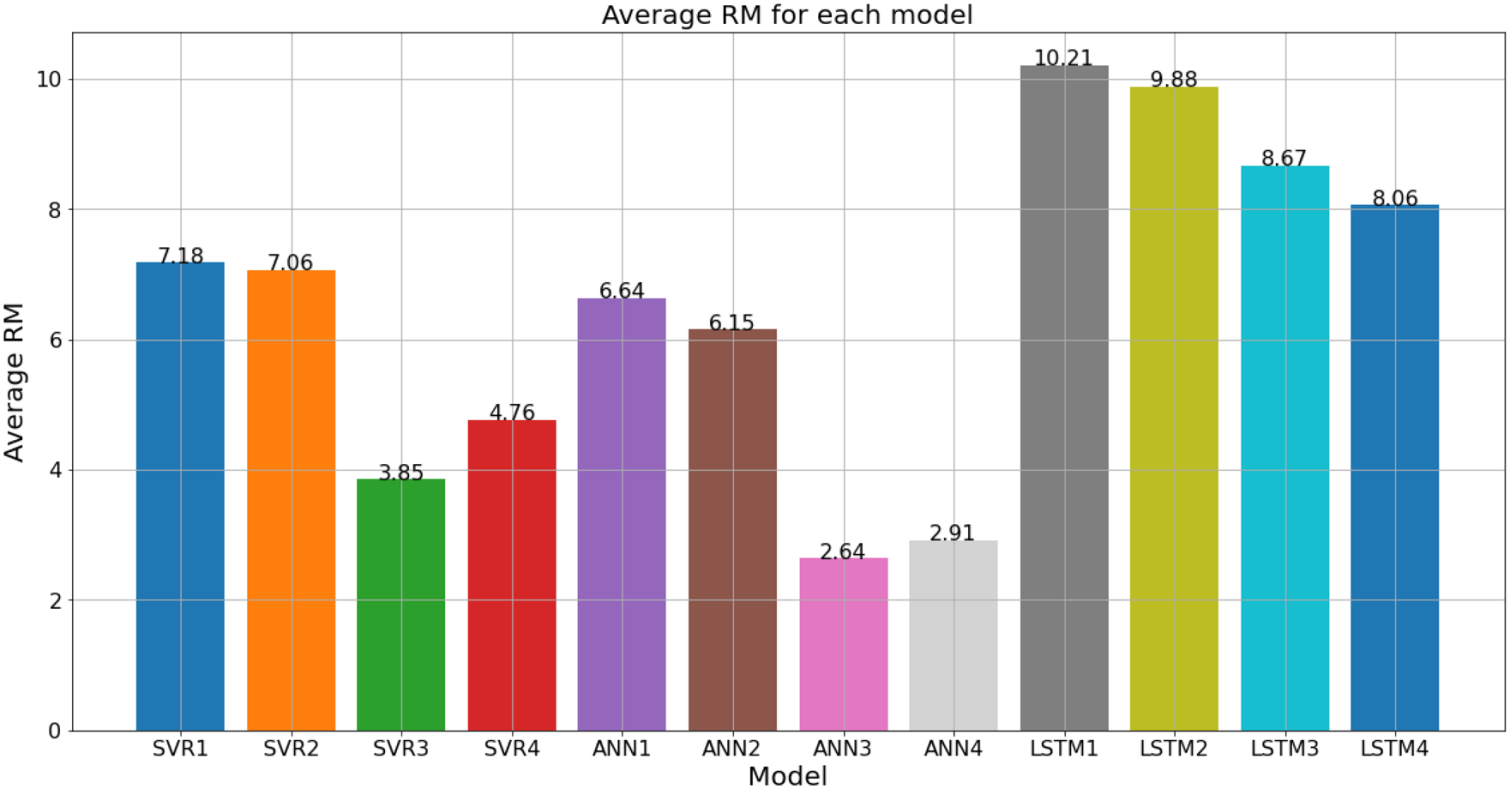
Bar chart of average RM for each model based on all data sets.

Next, the models’ performances are evaluated based on their reliability in producing relatively high-accuracy predictions for different data sets. This evaluation is important to determine the models that are most adaptable and robust to different data sets, which may vary in SSL magnitude and temporal behaviour. It also helps to understand each models’ overall performance on all 11 tested data sets. To quantify the models’ reliability in producing relatively high-accuracy predictions for different data sets, the average of the RM scores obtained by each model for all 11 tested data sets are calculated and compared, as shown in Table 23 and Fig. 28. It is found that ANN3 has the highest average RM with a score of 2.64, hence making it the most reliable model in predicting SSL with relatively high accuracy for different data sets. ANN4 is a close competitor (average RM = 2.91), followed by SVR3 (average RM = 3.85).

**Table 23:** Average RM of each model based on all data sets.

Data set	RM											
	**SVR1**	**SVR2**	**SVR3**	**SVR4**	**ANN1**	**ANN2**	**ANN3**	**ANN4**	**LSTM1**	**LSTM2**	**LSTM3**	**LSTM4**
Sungai Johor	8.67	6.33	7.00	8.00	6.67	3.67	1.00	2.00	11.00	12.00	6.00	5.67
Sungai Muda	9.33	8.33	3.00	3.67	6.00	4.67	1.00	2.67	10.67	10.33	8.67	9.67
Sungai Kelantan	7.67	6.67	2.33	4.00	6.67	5.00	1.67	2.00	11.33	11.33	10.33	9.00
Sungai Melaka	6.00	5.00	1.00	2.00	8.00	7.00	4.00	3.00	12.00	11.00	10.00	9.00
Sungai Kepis	5.67	4.67	4.33	3.67	10.00	12.00	2.67	5.00	5.00	6.00	9.00	10.00
Sungai Pahang	8.33	10.00	10.00	11.00	2.00	1.00	8.67	5.00	3.67	3.33	7.00	8.00
Sungai Perak	6.00	5.00	1.00	2.00	7.67	9.33	3.00	4.00	12.00	11.00	9.33	7.67
Sungai Arau	8.33	11.33	5.67	7.67	6.00	3.33	1.00	2.00	11.33	10.33	6.00	5.00
Sungai Selangor	5.33	7.00	3.00	4.00	6.67	7.00	2.00	1.00	11.33	11.67	10.00	9.00
Sungai Dungun	7.67	8.33	4.00	3.67	5.33	5.00	1.67	1.33	12.00	11.00	10.00	8.00
Sungai Klang,	6.00	5.00	1.00	2.67	8.00	9.67	2.33	4.00	12.00	10.67	9.00	7.67
Average RM	7.18	7.06	3.85	4.76	6.64	6.15	2.64	2.91	10.21	9.88	8.67	8.06

Based on these comparisons and analyses, it can be deduced that the best model for SSL prediction in the present case study of Peninsular Malaysia is the ANN3 model as it produced the best SSL predictions for more data sets compared to the other models, and it is the most reliable model given that it is robust and adaptable enough to predict SSL with a relatively high accuracy for different data sets compared to the other models, as suggested by its lowest average RM score of 2.64.

As highlighted by Table 21 and Fig. 28, it can be understood that ANN is the most successful algorithm in the present study, followed by SVR. LSTM represents the poorest performing algorithm as it was not able to produce the best predictive performance for any of the tested data sets. The LSTM models also have the lowest average RMs compared to the other models. Generally, LSTMs are effective in predicting based on data sets that have a clear time pattern. As the SSL data in the present study is volatile as it is often going up and down without a clear time pattern, it is probable that LSTM’s effectiveness may have been reduced. Meanwhile, SVR and ANN produced better SSL predictions because they are regression-based methods.

## Conclusion

Time series data sets on daily SF and SSL were obtained for 11 different rivers throughout Peninsular Malaysia and used to develop ML models for SSL prediction using three ML algorithms, namely SVM, ANN, and LSTM. Based on quantitative analyses, the ANN3 model, which utilises the ANN algorithm and input scenario 3 (inputs consisting of current day SF, previous day SF, and previous day SSL) is the best performing SSL-predicting model. ANN3 was able to produce the best predictive performance for the most data sets that were tested in the present study, which is 5 out of 11 data sets; and emerged as the most reliable model in predicting SSL with relatively high accuracy for different data sets. Analysis has also shown that the ANN algorithm and input scenario 3 were most successful as they were each able to produce the best predictions for 8 out of 11 data sets.

To conclude, the present study has contributed towards the testing and development of SSL predicting models for multiple rivers within Peninsular Malaysia, given that the development and proposal of predictive models based on multiple river data sets within a single study are scarce. This research gap has been addressed, and the main purpose of the present study which is the proposal of a single model that is capable of producing accurate SSL predictions for rivers within Peninsular Malaysia is achieved. Based on the findings, the present study proposes the ANN3 model as the model that has the best capability of producing accurate SSL predictions for rivers within Peninsular Malaysia. The present study is hoped to contribute towards the respective body of knowledge and help hydrological-related organisations in employing suitable and accurate models for SSL prediction. Future studies may focus on further improving the ANN3 model for SSL prediction in Peninsular Malaysia by hybridizing the model or incorporating more advanced techniques. Additionally, future studies may further study and test the ANN3 model on in other regions around the globe, to determine the effectiveness and accuracy of the ANN3 model on a larger scale. The method of selecting the best SSL predictive ML model in the present study, which involves using performance evaluation measures to determine the model that produces the best SSL predictions for the most rivers and obtains the best average RM, may also be further studied and tested on case studies within other regions.

## Data Availability

The data that support the findings of this study are available at the Malaysian Department of Irrigation and Drainage.
